# Dietary oxidized lipids in redox biology: Oxidized olive oil disrupts lipid metabolism and induces intestinal and hepatic inflammation in C57BL/6J mice

**DOI:** 10.1016/j.redox.2025.103575

**Published:** 2025-03-01

**Authors:** Yifan Bao, Magdalena Osowiecka, Christiane Ott, Vasiliki Tziraki, Lukas Meusburger, Claudia Blaßnig, Daniela Krivda, Petra Pjevac, Joana Séneca, Matthias Strauss, Christina Steffen, Verena Heck, Soner Aygün, Kalina Duszka, Kevin Doppelmayer, Tilman Grune, Marc Pignitter

**Affiliations:** a Institute of Physiological Chemistry, Faculty of Chemistry, University of Vienna, Vienna, Austria; b Doctoral School of Chemistry, Faculty of Chemistry, University of Vienna, Vienna, Austria; c Department of Molecular Toxicology, German Institute of Human Nutrition Potsdam-Rehbruecke, Nuthetal, Germany; d Joint Microbiome Facility of the Medical University of Vienna and the University of Vienna, Medical University of Vienna, University of Vienna, Vienna, Austria; e Department of Nutritional Sciences, Faculty of Life Sciences, University of Vienna, Vienna, Austria

**Keywords:** Oxidized olive oil, Inflammation, Lipid metabolic dysfunction, Gut microbiota, 9,10-Epoxy-stearic acid

## Abstract

Olive oil, rich in oleic acid, is often regarded as a healthier alternative to animal fats high in saturated fatty acids and plant oils rich in oxidizable polyunsaturated fatty acids. However, the redox biological implications and health effects of oxidized olive oil (ox-OO) remain underexplored. Our study investigated its impact on lipid metabolism, intestinal and hepatic inflammation, and gut microbiota. Female C57BL/6J mice were fed either a standard normal (NFD), high-fat diet (HFD), an NFD-ox-OO or HFD-ox-OO, in which ox-OO (180 °C heating, 10 min) was the sole lipid source. Inflammation was assessed using macrophage marker F4/80 immunohistochemical (IHC) staining. Gene expression of inflammatory and lipid metabolism markers (IL-10, NF-kBp65, IL-1β, TNFα, TLR4, COX2, PPARα, PPARγ, CPT1a, SCAD, MCAD, LCAD) was analyzed by qRT-PCR. Soluble epoxide hydrolase (sEH) protein expression was measured using IHC. Oxylipin and carnitine profiles were determined by LC-MS/MS. Gut microbiota was analyzed by 16S rRNA sequencing. Ox-OO disrupted redox homeostasis, leading to lipid metabolic dysfunction in the intestines and liver. In the duodenum and proximal jejunum, ox-OO decreased the levels of anti-inflammatory oxylipins and increased pro-inflammatory mediators, leading to inflammation. In the ileum and colon, ox-OO caused lipid metabolic dysregulation and inflammation. Colon inflammation was linked to inhibited mitochondrial β-oxidation and decreased short-chain fatty acid-producing microbiomes. Notably, redox imbalances were further implicated by the identification of 9,10-epoxy-stearic acid, a novel inflammatory lipid mediator oxidized from dietary oleic acid, which upregulated sEH. Ox-OO affects lipid metabolism and may contribute to inflammation in the gut and liver, raising questions about the assumption that olive oil is always beneficial and suggesting possible risks linked to oxidized oleic acid.

## Highlights

• Lipid oxidation diminishes the health benefits olive oil and may pose health risk.
• Oxidized olive oil disrupts lipid metabolism and gut microbiota, causes inflammation.
• Oleic acid derived 9,10-epoxy-stearic acid upregulated soluble epoxide hydrolase (sEH).
• 9,10-epoxy-stearic acid is proposed as a novel dietary inflammatory lipid mediator.

## Graphical abstract

**Figure:**
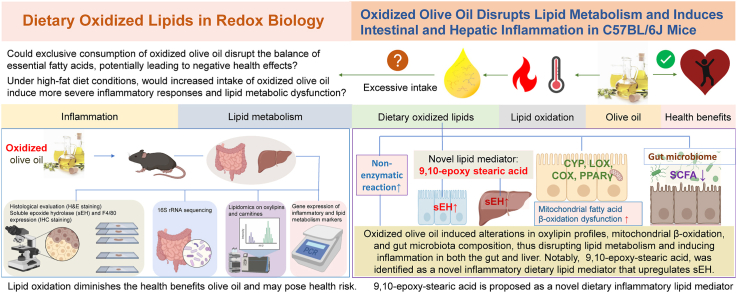

## 1 Introduction

Dietary lipids play a crucial role in intestinal health. Saturated fatty acids, particularly those found in animal fats such as pork lard, have been associated with intestinal inflammation and obesity-related disease [1]. Consequently, plant oils, rich in unsaturated fatty acids, have been promoted as a healthier alternative. However, the health effects of oxidized lipids—formed during storage or heating—remain a topic of debate. Earlier studies suggested oxidized plant oils caused minimal health impact in long-term animal studies [2], while recent investigations have challenged this notion, suggesting that oxidized oils may pose significant health risks. Dietary oxidized lipids derived from polyunsaturated fatty acids (PUFA) have been shown to promote the generation of reactive oxygen species and subsequently increase oxidative stress [3], regulate lipid mediators, influence lipid metabolism, and alter the gut microbiome [4], thus potentially inducing inflammation [5]. Additionally, recent research indicates that dietary oxidized lipids can significantly affect the gut-liver axis, thereby influencing lipid metabolism and triggering inflammatory responses in the liver [6].

Oxidized lipids derived from PUFAs, especially ω-6 PUFAs linoleic acid (LA) and arachidonic acid (ARA), are involved in inflammation and various other biological processes. PUFAs oxidized by enzymes such as lipoxygenases (LOX), cyclooxygenases (COX), and cytochrome P450 (CYP) oxidases, generate oxylipins, including hydroxy fatty acids, epoxy fatty acids, prostaglandins, and thromboxanes [7]. These oxylipins can be produced endogenously, but can also be obtained through dietary intake. Consequently, oxidized dietary lipids may act as lipid mediators, directly promoting inflammation or modulating endogenous fatty acid metabolism, thereby influencing oxylipin production and subsequent inflammatory responses. Furthermore, emerging studies have demonstrated that oxidized linoleic acid disrupted catabolic fatty acid metabolism [8], by influencing peroxisome proliferator-activated receptor α (PPARα) [9], carnitine palmitoyltransferase (CPT), and acylcarnitines, leading to an imbalance in intracellular carnitine homeostasis [10]. This imbalance subsequently affects the activity of long-chain acyl-CoA dehydrogenase (LCAD), medium-chain acyl-CoA dehydrogenase (MCAD), and short-chain acyl-CoA dehydrogenase (SCAD), impairing mitochondrial β-oxidation, which in turn regulate oxylipins metabolism and inflammation response in the intestines [11].

Recent research has confirmed the potential health risks of oxidized ω-6 PUFA on lipid metabolism and inflammation [3]. Hence, current trends suggest reducing ω-6 PUFA intake and increasing the consumption of monounsaturated fatty acid enriched oils, which are more resistant to oxidation [12]. Olive oil, favored for its high oleic acid content and bioactive compounds, may oxidize due to long-term storage or heating. Notably, oleic acid is prone to epoxidation, and epoxidized oleic acid (9,10-epoxy stearic acid) has been shown to induce oxidative stress in HepG2 cells [13]. Furthermore, epoxidized fatty acids can be absorbed into human body [14] and may act as lipid mediators involved in colon inflammation, with soluble epoxide hydrolase (sEH) proposed as a therapeutic target for colonic inflammation [15]. Consequently, dietary 9,10-epoxy stearic acid may also regulate lipid metabolism and inflammation, as a lipid mediator. In addition to the potential risks posed by epoxidized and oxidized oleic acid, the unsaturated fatty acids PUFAs in olive oil can also be oxidized during heating and digestion. Prolonged exposure to these oxidized lipids could affect intestinal inflammation, gut microbiota, and systemic lipid metabolism.

**Fig. 1:**
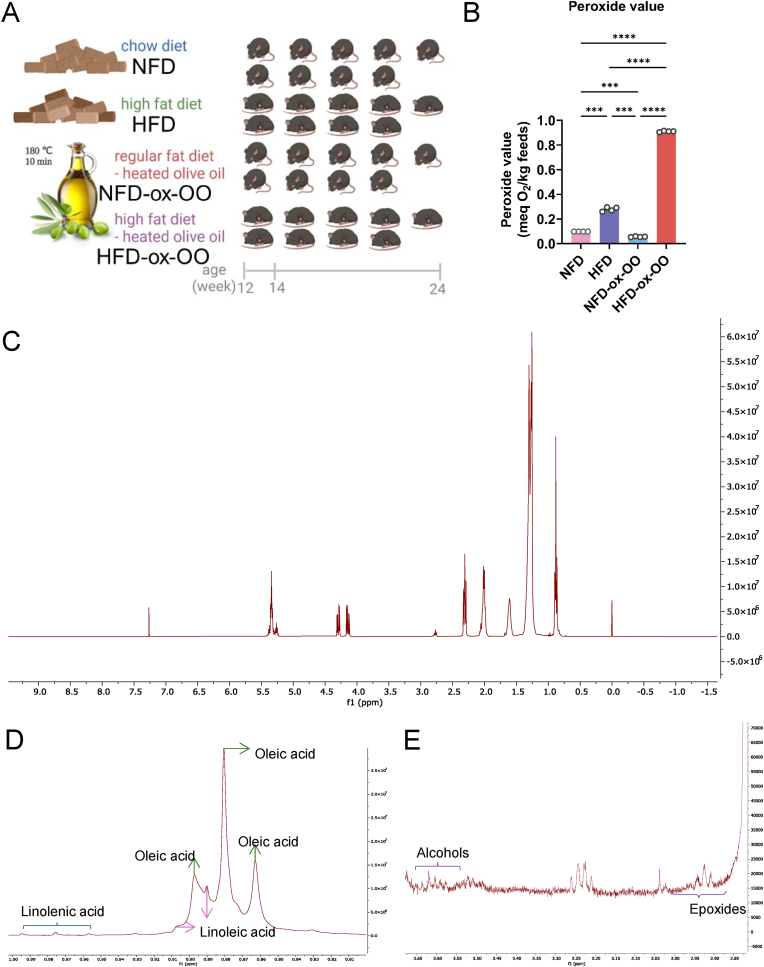
Lipid oxidation in experimental diets. (A) Experimental design for the animal study. (B) Peroxide values of diets. (C) ^1^H NMR spectra of lipid extracts from HFD-ox-OO. (D) Identification of unsaturated fatty acids based on ^1^H NMR chemical shifts of the –CH_3_ group. (E) ^1^H NMR spectral signals corresponding to epoxides and alcohols. Data are presented as mean or mean ± SEM. Statistical significance was determined by ANOVA followed by Tukey's post hoc test. ∗p < 0.05, ∗∗p < 0.01, ∗∗∗p < 0.001, ∗∗∗∗p < 0.0001.

While extensive research has explored the health benefits of olive oil, the effects of oxidized olive oil on lipid metabolism and inflammation remain underexplored, with no current scientific nutritional guidance regarding oxidized olive oil. To address this gap and provide molecular and biochemical insights, we fed C57BL/6J mice standard normal-fat and high-fat diets with oxidized olive oil (180 °C, 10 min) as the sole lipid source, NFD-ox-OO and HFD-ox-OO, as well as standard normal fat diet (NFD) and high-fat diet (HFD) as control (Fig. 1A). The main lipid source in the NFD was soybean oil, rich in ω-6 fatty acids (Table S1), while the HFD used lard as its main lipid source, rich in saturated fatty acids and containing a limited amount of oleic acid. The peroxide values revealed relatively low levels of oxidation in both NFD groups, with NFD-ox-OO showing an even lower peroxide value than NFD. In contrast, both HFD groups exhibited higher peroxide values, with the highest in HFD-ox-OO (Fig. 1B). This leads to two key questions.

(1) Could exclusive consumption of oxidized olive oil disrupt the balance of essential fatty acids, potentially leading to negative health effects?
(2) Under high-fat diet conditions, would increased intake of oxidized olive oil induce more severe inflammatory responses and lipid metabolic dysfunction than saturated fatty acids, e.g., lard?

To address these questions, we examined the effects of these diets on lipid metabolism, inflammatory responses in the intestines and liver, and changes in gut microbiota. Our study aims to observe early-stage intestinal inflammation induced by oxidized olive oil and elucidate underlying mechanisms, reassessing its health benefits., thus offering valuable insights into the broader health impact of dietary habits of heated olive oil.

## 2 Materials and methods

### 2.1 Lipid profile of oxidized olive oil

Extra virgin olive oil sourced from Croatia was heated to 180 °C to obtain oxidized olive oil (ox-OO), which was then used as the sole lipid source to formulate a custom diet for the mice (Table 1).

**Table 1:** Nutritional compositions of diets.

Ingredient	Unit	NFD	HFD	NFD-OO	HFD-OO
Casein	%	20.00	20.00	20.00	20.00
L-Cystin	%	0.25	0.25	0.25	0.25
Corn starch	%	43.5	17.45	43.4	17.40
Sucrose	%	15.00	10.00	15.00	10.00
Maltodextrin	%	5.00	5.00	5.00	5.00
Cellulose powder	%	5.00	5.00	5.00	5.00
Vitamin premix	%	1.00	1.00	1.00	1.00
Mineral premix	%	6.00	6.00	6.00	6.00
Choline Cl	%	0.25	0.25	0.25	0.25
Soybean oil	%	4.00	–	–	–
Pork lard	%	–	35.00	–	–
Olive Oil	%	–	–	4.00	35.00
Food dyes	%	–	0.05	0.10	0.10
**Proximate contents**	**%**				
Crude protein	%	17.7	17.7	17.7	17.7
Crude fat	%	4.1	35.1	4.1	35.1
Crude fiber	%	5.0	5.0	5.0	5.0
Crude ash	%	5.4	5.4	5.4	5.4
Starch	%	41.8	16.8	41.7	16.7
Sugar	%	6.0	6.0	6.0	6.0
Dextrin	%	14.8	14.8	14.8	14.8
**ME (Atwater)**	MJ/kg	15.2	21.9	15.2	21.9
Protein	kJ%	19.5	13.5	19.5	13.5
Fat	kJ%	10.3	60.5	10.3	60.5
Carbohydrates	kJ%	70.3	26.0	70.3	26.0

#### 2.1.1 Peroxide value

Considering potential further oxidation during the feed processing, lipids were extracted from the diet [16] for further analyses. The peroxide value was then measured using an spectrophotometric method adapted from Ref. [16]. 5 g of feed was dissolved in 50 mL chloroform/methanol (2:1 v/v) containing 0.1 % BHT, then rotated for 1 h under argon, and filtered into a separation funnel. Subsequently, 10 mL of KCl solution was added and mixed with the sample. After 10 min, the organic phase was dried under a nitrogen and weighed.

15–25 mg of lipid extract or standard (0.2–3 mg/L Iron (III) chloride solution) was dissolved in 9.9 mL of chloroform/methanol (7:3 v/v), treated with 50 μL of ammonium thiocyanate (0.3 g/mL) and 50 μL of iron (II) chloride solution. The mixture was vortexed and incubated at room temperature for 5 min. After incubation, approximately 1 mL of the treated sample was transferred to a quartz cuvette, and the absorbance was measured at 500 nm. The peroxide value was calculated as the following formula:

$$POV\;\left(meq\;O_{2}/kg\;sample\right)=\frac{\left(A_{500S}-A_{500B}\right)∙1/k}{55.84∙m∙2}$$

where A500_S_ and A500_B_ are the absorbances at 500 nm of samples and the blank, respectively; k is the slope of the calibration curve, 55.84 is the atomic weight of iron, m is the initial weigh of the feeds sample (g) and 2 is the conversion factor between oxygen milliequivalents and peroxide milliequivalents.

#### 2.1.2 ^1^H NMR analysis

Lipids were extracted from feed samples using dichloromethane as previously described [17]. Briefly, 100 mg of feed was mixed with 125 μL of dichloromethane and 1.25 μL of HCl, vortexed for 2 min, and centrifuged at 16,000g for 20 min. The organic phase was collected, and the extraction was repeated six times. The pooled extracts were evaporated under nitrogen and dissolved in 600 μL of deuterated chloroform containing 0.1 % TMS as an internal reference for ^1^H NMR analysis. ^1^H NMR spectra of lipid extracts were acquired in triplicate using an Avance 400 spectrometer (Bruker, Billerica, MA, USA) operating at 400 MHz, following the method described in Ref. [18]. Spectra were processed using MestreNova software (Mestrelab Research, Santiago de Compostela, Spain).

### 2.2 Mice study

Wild-type female C57BL/6J mice (eight-week-old) were randomly allocated into four groups (n = 9 mice/group) following a two-week period of acclimatization to their respective diets. The dietary interventions include: NFD, HFD, normal or high fat diet with oxidized olive oil (180 °C, 10 min) as the only dietary lipid source, NFD-ox-OO and HFD-ox-OO (Fig. 1A, Table S1). Mice were provided *ad libitum* access to food and water, subjected to a 12-h light/dark cycle, maintained at a constant room temperature of 22 ± 2 °C, and housed with access to a running wheel in each cage. After 12 weeks of dietary intervention, mice were humanely euthanized via isoflurane inhalation followed by cervical dislocation, and liver, white adipose tissue and intestinal tissues (duodenum, proximal and distal jejunum, ileum, cecum, and colon) and colon contents were collected, snap-frozen in liquid nitrogen and stored at −80 °C or fixed in 4 % paraformaldehyde at room temperature for 24 h for further analyses.

This mice study was carried out at the German Institute fo Human Nutrition, Potsdam-Rehbruecke, Germany, following the ARRIVE guidelines and the European Directive 2010/63/EU, approved by the competent authorities (Landesamt für Arbeitsschutz, Verbraucherschutz und Gesundheit Land Brandenburg; approval number: TVA-Nr: 2347-21-2021).

### 2.3 Histological analysis

#### 2.3.1 Hematoxylin and eosin staining (H&E)

Fresh tissue samples were fixed in 4 % paraformaldehyde at room temperature for 24 h, washed with PBS, and then embedded in paraffin. The 2 μm paraffin sections were deparaffinized using Roti®-Histol (Carl Roth) and rehydrated through an ethanol gradient (100–40 %). For H&E staining, hematoxylin solution (Sigma-Aldrich) was applied for 45 s, followed by a 10-s rinse with tap water, and then eosin (Sigma-Aldrich) for 1 min. After staining, the slides were mounted with Entellan® (VWR), and scanned Axio Scan 7 (Zeiss, Oberkochen, Germany), and analysis was performed using Zeiss ZEN 3.0 Blue Edition.

#### 2.3.2 Immunohistological chemistry (IHC)

IHC was performed using the Mouse and Rabbit Specific HRP/DAB Detection IHC kit (Abcam) following the manufacturer's instructions with minor modification.

Tissue sections were deparaffinized by washing in xylene for 5 min for twice, followed by ethanol washes at 100 %, 95 %, and 70 % for 10 min each, and a final 5 min wash in water. Antigen retrieval was achieved by heating sections in 0.01 M citrate buffer (pH 6.0) at 95 °C for 10 min, followed by 30 min cooling, then washing three times in water (5 min each). Tissue slides were then incubated with Hydrogen Peroxide Block for 10 min, followed by three 5 min TBST washes. After a 10 min Protein Block incubation and another set of three 5 min TBST washes, the slides were incubated overnight at 4 °C with 1:100 Goat F(ab) Anti-Mouse IgG H&L in 0.3 % BSA, followed by three TBST washes. Subsequently, slides were incubated with primary antibodies as shown in Table S1, followed by four 5 min TBST washes. After 10 min incubation of Biotinylated Goat Anti-Polyvalent secondary antibody, slides were washed with TBST four times for 5 min each. Streptavidin Peroxidase was applied for 10 min, followed by four TBST washes. DAB solution was applied for 10 min, followed by four TBST rinses. Hematoxylin Gill counterstaining was followed by four water rinses and graded ethanol washes (5 min each in 70 %, 95 %, and 100 %). Stained samples were sealed with Entellan™ (Sigma-Aldrich) and coverslipped.

Microscopy was performed on Vectra Polaris™ Automated Quantitative Pathology Imaging System with brightfield whole-slide scanning at 20 × magnification. Staining intensities of sEH and F4/80 were quantified using HALO Software. Tissue layers were classified using random forest machine learning, and DAB-stained areas were quantified with Indica Labs Area Quantification v2.4.3.

### 2.4 Gene expression analysis in the tissue samples

Intestinal tissue total RNA extraction was performed using the Monarch® Total RNA Miniprep Kit, followed by cDNA synthesis using the LunaScript® RT SuperMix Kit. Subsequently, real-time quantitative PCR (qPCR) was conducted using Luna® Universal qPCR Master Mix and primers detailed in Table S2. The expression levels of target genes were normalized to the housekeeping genes 18S and GAPDH (intestines), HPRT and GAPDH (liver and white adipose tissue).

### 2.5 Targeted metabolomics

#### 2.5.1 Semi-quantification of oxylipins based on LC-MS/MS

Oxylipins were extracted from tissues using a modified solid phase extraction (SPE) method based on Dumlao's protocol [19]. Tissue samples (20–30 mg) were mixed with 1.5 mL of 10 % methanol, 20 μL of standard mix (Table S3), and ten homogenization ceramic beads, then homogenized at 4000 rpm for 4 min. Subsequently, SPE columns (StrataTM-x, 33 μm Polymeric Reversed Phase, 60 mg/30 mL) were activated with 3 mL of 100 % methanol and 3 mL of bidistilled water. The homogenized samples were loaded onto the columns, washed with 3 mL of 10 % methanol, and oxylipins were eluted with 1 mL of methanol. Samples were then dried under nitrogen and reconstituted with 100 μL of methanol for LC-MS/MS analysis.

The HPLC system (Shimadzu Prominence LC-20AD) was coupled to a Shimadzu LCMS8040 mass spectrometer via an ESI interface. Separation was performed on a Phenomenex Kinetex C18 column (150 mm × 2.1 mm, 2.6 μm, 100 Å) with the column oven set to 40 °C and an injection volume of 20 μL. Solvent A consisted of 95 % H_2_O and 5 % acetonitrile with 0.1 % acetic acid and 10 mM ammonium acetate, while Solvent B consisted of 95 % acetonitrile and 5 % H_2_O with 0.1 % acetic acid and 10 mM ammonium acetate. The gradient elution was as follows: 0–2 min at 5 % B, 14 min at 70 % B, 24–29 min at 95 % B, followed by re-equilibration at 5 % B for 10 min, with a flow rate of 0.3 mL/min. ESI-MS conditions included a nebulizing gas flow of 3 L/min, drying gas flow of 10 L/min, desolvation line temperature of 150 °C, heating block temperature of 350 °C, and interface bias of −3.5 kV. CID gas was Ar at 230 kPa. The mass spectrometer was operated in MRM mode, with transitions for standards and oxylipins as shown in Table S3 and Table S4.

#### 2.5.2 Semi-quantification of carnitines based on LC-MS/MS

The extraction of carnitines from tissue samples was conducted using a modified biphasic solvent system based on [20], the extracted polar phase was derivatized to obtain butylated compounds, particularly acylcarnitines, following the procedure described in Ref. [21].

Briefly, 225 μL of ice-cold methanol and 10 μL of a 1 μM carnitine internal standard (Table S5) mix were mixed with 20–25 mg tissue samples and 10 ceramic beads (1.4 mm), and homogenized for 2 min at 2500 rpm. Subsequently, 750 μL ice-cold MTBE were added and vortexed for 10 s, followed by shaking at 4 °C for 6 min. Then, 188 μL LC-MS grade water was added and vortex for 20 s, and centrifuged for 2 min at 14000 rpm at 4 °C.

Subsequently, 100 μL butanol (5 % v/v acetyl chloride) was mixed with the polar phase and vortexed for 10 s. After incubation at 60 °C for 20 min, samples were dried in a vacuum concentrator. The samples containing butylated acylcarnitines were reconstituted in 50 μL acetonitrile/water (80:20, v/v) and vortexed for 10 s. 40 μL of the supernatant were transferred into LC vials for measurement.

Measurement of carnitines was conducted using a Shimadzu LCMS 8040, including an LC-20AD pump, a SIL-20AC autosampler at 4 °C and a CTO-20AC oven with a Phenomenex Kinetex 2.6 μm HILIC 100 Å, LC Column (150 × 2.1 mm) and an ESI interface. Data acquisition was performed in positive multiple reaction monitoring mode. The interface was run with a nebulizing gas flow of 3 L/min, a drying gas flow of 10 L/min, a desolvation line temperature of 150 °C, and a heat block temperature of 350 °C. Solvent A contained 10 mM ammonium acetate with 0.2 % formic acid in water and solvent B was acetonitrile. 2 μL of samples were injected and eluted with a flow rate of 0.5 mL/min with the following gradient: 0–5 min, 90%-80 B, 5–10 min, hold at 80 % B, 10.0–10.1 min, 80–90 % B. The oven temperature was at 40 °C. Dwell time was 75 ms and the collision energy was adjusted according to the sources used for the MRM approach. The MRM transitions are detailed in Table S6.

The chromatograms were analyzed using Skyline (64-bit) v.23.1.0.380. Oxylipins and carnitines were quantified by relating the area of the sample to the area of the internal standard, multiplied by the internal standard concentration.

### 2.6 16s RNA sequencing of gut microbiota

#### 2.6.1 DNA extraction, 16S rRNA gene amplicon preparation and sequencing

DNA extraction from mice fecal pellets was performed with the QIAamp Fast DNA Stool Mini Kit (Qiagen, Germany), and the V4 region of bacterial and archaeal 16S rRNA genes was amplified with the primer pairs 515F/806R6,7. Amplification was performed following a standardized 2-step PCR protocol, described in detail in Ref. [22], and amplicons were sequenced on the Illumina MiSeq platform (v3 chemistry, 600 cycles, 2 x 300bp). Amplicon pools were extracted from the raw sequencing data using the FASTQ workflow in BaseSpace (Illumina) with default parameters.

#### 2.6.2 16S rRNA gene amplicon data processing

Demultiplexing of amplicon libraries was performed with the python package demultiplex (Laros JFJ, github.com/jfjlaros/demultiplex), allowing for one mismatch to barcode and two mismatches for linker and primer sequences. Amplicon sequence variants (ASVs) were inferred using the DADA2 R package v1.429 with the recommended workflow.10 FASTQ reads 1 and 2 were trimmed at 220 nt and 150 nt, respectively, allowing for two expected errors, and inferred ASV sequences were classified using DADA2 and the SILVA database SSU Ref NR 99 v138.111 with a confidence threshold of 0.5. ASVs without classification and ASVs classified as eukaryotes, mitochondria, or chloroplasts, as well as known reagent contaminants were filtered before downstream analysis. Only samples with at least 1000 read pairs after filtering were kept for further analyses.

#### 2.6.3 16S rRNA gene amplicon analyses

16S rRNA gene amplicon analyses were performed using R v4.3.2 and Bioconductor v3.16 packages SummarizedExperiment v1.32, SingleCellExperiment v1.24, TreeSummarizedExperiment v2.8 [23] mia v1.8 (https://github.com/microbiome/mia), vegan v2.6-4 (https://CRAN.R-project.org/package=vegan), phyloseq v1.44 [24], microbiome v1.22 (http://microbiome.github.io), DESeq2 v1.38.3 and microViz v0.10.8 [25]. Alpha diversity (i.e. richness and diversity indexes) was calculated on rarified data using R packages vegan and mia. Beta diversity was calculated by performing a PCoA using the Aitchison distance, with R package microViz. The difference in per-group centroids was tested with a PERMANOVA on Aitchison distances using R packages vegan and microViz. Pairwise differential abundance testing was performed using DESeq2 with genus-level clustered log-transformed relative abundance data, after adding a pseudocount of 1.

### 2.7 Statical analysis

Statistical data analysis was undertaken using Prism 9 for Windows 64-bit v9.4.0 (GraphPad Software, LLC). Outliers were identified using the ROUT method (Q = 1 %). Shapiro-Wilk test (α = 0.05) was performed to check for normal distribution. F-test (p < 0.05) was employed to investigate homoscedasticity. For comparing two normally distributed and homoscedastic data groups, unpaired t-tests (p < 0.05) were performed, while Welch's correction was applied for data with unequal variances. In cases where the data groups were not normally distributed, the Mann-Whitney test (p < 0.05) was utilized. When comparing three or more groups, variances were assessed using the Brown-Forsythe test (p < 0.05). For normally distributed groups with equal variances, an ordinary one-way ANOVA (p < 0.05) was employed to detect significant differences among means. Multiple comparisons were tested with Tukey's post-hoc test. In instances of unequal variances, the Brown-Forsythe ANOVA (p < 0.05) with Dunnet's T3 multiple comparisons test was performed. If the data were not normally distributed, significance was determined via the Kruskal-Wallis test (p < 0.05) followed by Dunn's post hoc test. All post-hoc tests were performed with α = 0.05.

## 3 Results

### 3.1 Lipid profile of oxidized olive oil

Lipids were extracted from the animal feeds, and peroxide values were measured (Fig. 1B). HFD-ox-OO exhibited a higher hydroperoxide content, indicating greater lipid oxidation than the other diets. To further characterize lipid oxidation in HFD-ox-OO, extracted lipids were analyzed by ^1^H NMR spectroscopy (Fig. 1C). The major unsaturated fatty acids identified included oleic, linoleic, and linolenic acids (Fig. 1D). In addition to primary oxidation products such as hydroperoxides (Fig. 1B), H NMR also detected secondary oxidation products, including alcohols and epoxides [26], which might be dereived from oleci, linoleic and linolenic acids.

### 3.2 Histological and morphological effects of oxidized olive oil on the intestines

**Fig. 2:**
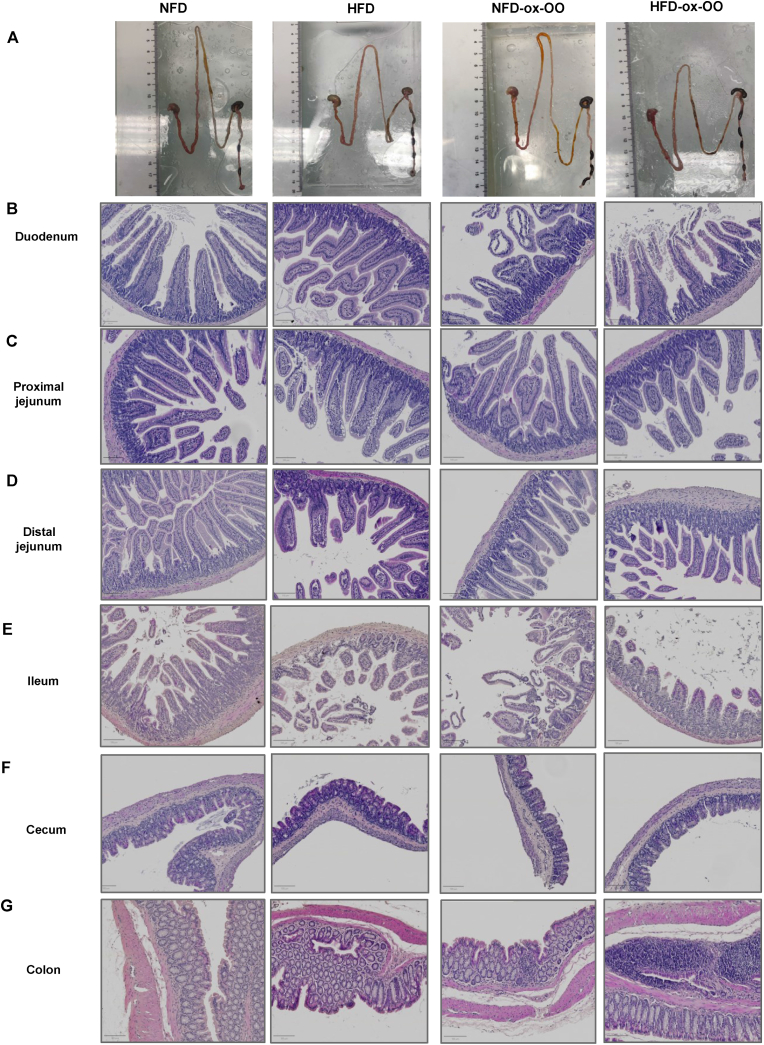
Histological and morphological effects of oxidized olive oil on the intestines. (A) Photographs of the gastrointestinal tract of mice. Representative H&E-stained sections and histological scores of (B) duodenum, (C) proximal jejunum, distal jejunum, (E) ileum, (F) cecum, and (H) colon.

Both HFDs resulted in a shortened small intestine length (Fig. 2A), indicating that heated olive oil may also have potential negative effects on the intestine [27]. Histological evaluation of the duodenum (Fig. 2B) and proximal jejunum (Fig. 2C) revealed significant epithelial damage, architectural changes, overall thinning of the intestinal wall and immune cell infiltration in the HFD, ox–OO–NFD, and ox–OO–HFD groups compared to the NFD group. Similar trends were observed in the cecum (Fig. 2F). Notably, in ileum (Fig. 2E) and colon (Fig. 2G), the terminal sections of the small and large intestines, showed the most pronounced alterations for the ox–OO–NFD and ox–OO–HFD groups, including damaged epithelium, shortened villi, and mild erosion and ulceration. The lamina propria was densely infiltrated with mononuclear cells and polymorphonuclear cells, particularly neutrophils. Additionally, as in the HFD group, notable lipid droplet accumulation can be also observed in HFD-ox-OO group, especially in the proximal jejunum. These findings suggest inflammatory response and potential effects on lipid metabolism within the intestines. Interestingly, in the distal jejunum (Fig. 2D), which is exposed to dietary factors for the longest duration, ox-OO treatment showed minimal impact on intestinal morphology.

### 3.3 Effects of oxidized olive oil on the inflammatory responses in the intestines

**Fig. 3:**
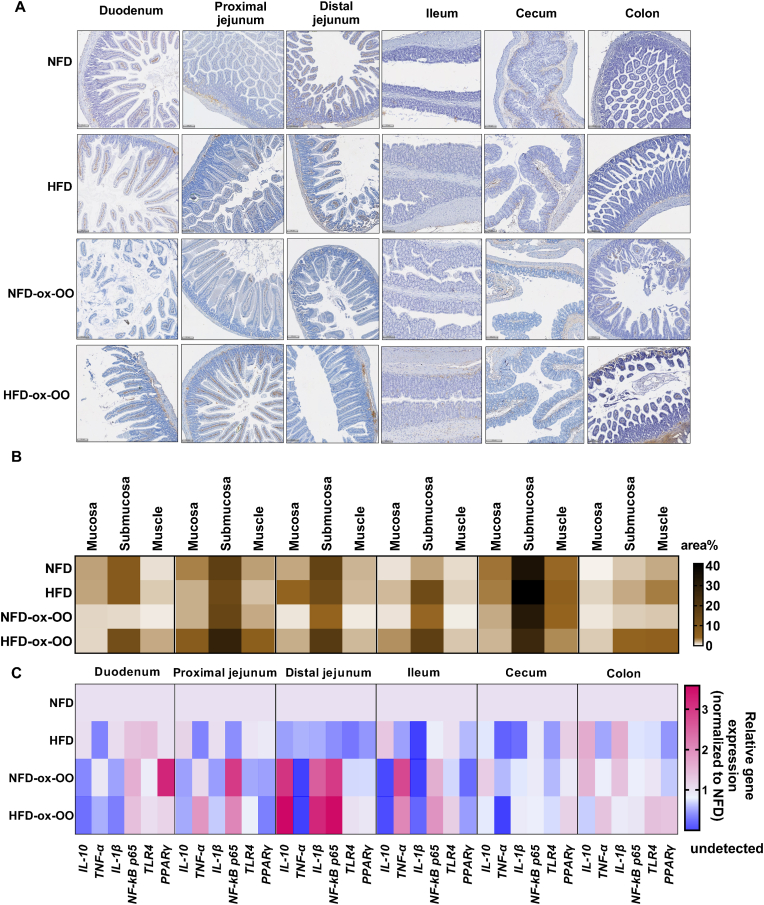
Effects of oxidized olive oil on the inflammatory responses in the intestines. (A) Representative tissue sections showing F4/80 immunostaining in intestines. (B) Quantification of the area% of F4/80 in mucosa, submucosa, and muscles in the intestinal sections. (C) Gene expression levels of IL-10, TNF-α, IL-1β, NF-kB p65, TLR4 and PPARγ in the intestines. Data are presented as mean.

To investigate the impact of ox-OO on intestinal inflammation, F4/80 macrophages in intestine were evaluated (Fig. 3A). Regardless of diet, F4/80 expression was most prominent in the submucosa of the small intestine. The HFD-ox-OO group exhibited the highest F4/80 expression in the duodenum submucosa (Fig. S1A) compared to NFD, HFD and NFD-ox-OO. Notably, in the proximal jejunum, F4/80 expression was highest in the HFD-ox-OO (Fig. S1B). The distal jejunum showed the most significant pro-inflammatory response in the HFD group, with the highest F4/80 expression in the mucosa (Fig. S1C). Conversely, F4/80 expressed lower in the NFD-ox-OO group, consistent with morphological observations. In the ileum, F4/80 expression was highest in the HFD-ox-OO group, particularly in the mucosa (Fig. S1D). F4/80 levels were highest in the submucosa of the cecum compared to other sections (Fig. 3B), but diet had minimal impact. In the colon, F4/80 expression was consistently most pronounced in the HFD-ox-OO group (Fig. S1E), indicating a strong pro-inflammatory effect.

After feeding with NFD-ox-OO and HFD-ox-OO, inflammatory regulations were observed in the duodenum, proximal jejunum, distal jejunum, ileum, and colon, with minimal changes in the cecum (Fig. 3C, Fig. S1G-L). In the duodenum, ox-OO significantly reduced anti-inflammatory IL-10 gene expression (Fig. 3C, Fig. S1G). In the proximal jejunum, IL-10 expression decreased while TNF-α increased, indicating an inflammatory response (Fig. 3C, Fig. S1H). In the distal jejunum, ox-OO upregulated IL-10 gene expression, consistent with pathological observations (Fig. 3C, Fig. S1I). Notably, IL-10 showed a tenfold decrease, and NF-kB p65 was upregulated in the ileum (Fig. 3C, Fig. S1J), indicating a severe inflammatory response. In the colon, IL-10 was downregulated, and TLR4 was significantly upregulated in HFD-ox-OO compared to NFD-ox-OO (Fig. 3C, Fig. S1L), suggesting altered colonic microbiota and increased inflammation.

Ox-OO diets regulated PPARγ expression in the intestines (Fig. 3C). NFD-ox-OO upregulated PPARγ in the duodenum, while HFD-ox-OO downregulated it (Fig. 3C, Fig. S1G), indicating high oxidized lipid levels induce inflammation. PPARγ was significantly suppressed in the proximal jejunum (Fig. 3C, Fig. S1H) and ileum (Fig. 3C, Fig. S1J) in both groups. Notably, PPARα was also reduced in the proximal jejunum, suggesting even small oxidized lipid amounts may cause inflammation and metabolic dysfunction. Interestingly, unchanged PPARγ expression in the distal jejunum further suggests ox-OO does not induce a pro-inflammatory response there (Figs. 1D and 3).

### 3.4 Effects of oxidized olive oil on intestinal fatty acid oxygenation

**Fig. 4:**
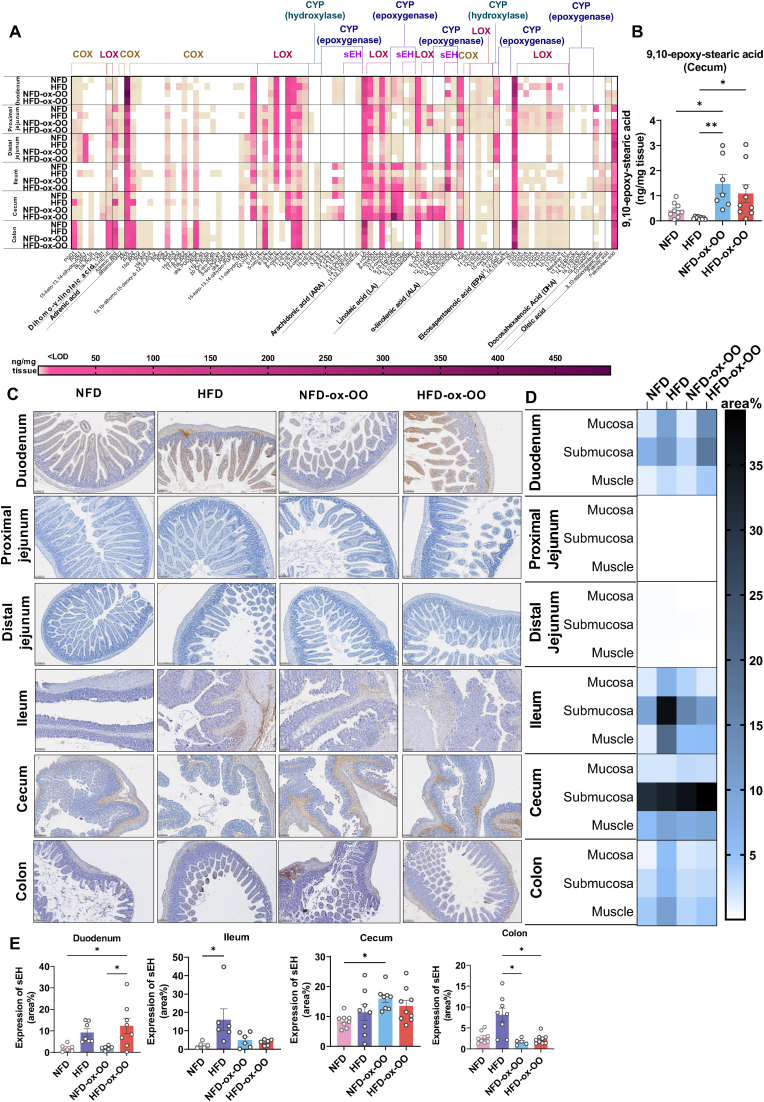
Effects of oxidized olive oil on intestinal lipid oxygenation. (A) Oxylipin concentrations in intestines. (B) 9,10-epoxysteric acid in cecum. (C) Representative tissue sections showing sEH immunostaining in intestines. (D) Quantification of the area% of sEH in mucosa, submucosa, and muscles in the intestinal sections. (E) Area% of sEH in duodenum, ileum, cecum and colon. Data are presented as mean or mean ± SEM. Statistical significance was determined by ANOVA followed by Tukey's post hoc test. ∗p < 0.05, ∗∗p < 0.01.

The composition of fatty acids in the diet has been shown to influence the levels of essential fatty acids in the body, and the oxidation products of these essential fatty acids can act as lipid mediators to regulate inflammatory responses, for instance, as ligands for PPARγ, IL-10 [28]. Therefore, we investigated the concentrations of oxylipins derived from dihomo-γ-linoleic acid (DGLA), adrenic acid, ARA, LA, α-linolenic acid (ALA), eicosapentaenoic acid (EPA) and docosahexaenoic acid (DHA) via COX, LOX, and CYP pathways, as well as the oxidation products of oleic acid, including 9,10-epoxy-stearic acid and 10-nitrooleate, and palmitic acid and palmitoleic acid in the intestines (Fig. 4A).

Significant regional differences in essential fatty acid levels within the intestine were observed (Fig. 4A, Figs. S2A–B). LA was lowest in the colon, ALA was reduced in the distal jejunum and ileum, while adrenic acid increased from the upper to the lower intestine, whereas ARA decreased. EPA levels were lowest in the cecum, and DHA was highest in the duodenum and colon. ARA-derived oxylipins were mainly formed by LOX than by COX, particularly in the duodenum, jejunum, and colon, while they were more epoxidized by CYP in the ileum and cecum. LA metabolism followed a similar pattern. ALA-derived oxylipins were relatively low in the colon and distal jejunum, with most ALA-derived LOX oxylipins undetectable. EPA- and DHA-derived oxylipins were relatively low, with these derivatives nearly undetectable in the distal jejunum. DHA-derived oxylipins were present in the proximal jejunum and cecum. Additionally, CYP-derived oxylipins and their sEH-hydrolyzed products were extremely low in the jejunum, consistent with the low sEH expression (Fig. 4C and D). Additionally, oleic acid metabolites were detected from the ileum onward.

Oxylipin profiles varied significantly with diets, showing marked differences across dietary groups (Figs. S2C–D). In the duodenum, diet significantly influenced oxylipin concentrations (Fig. 4A, Figs. S3A–B). HFD significantly influenced DGLA metabolism (Figs. S3C–D). Notably, COX2 expression was highest in the HFD group (Fig. S3E), corresponding with the upregulation of COX-ARA-derived prostanoids (Figs. S3F–G). HFD also enhanced ARA metabolism via LOX pathways (Fig. S3H), inducing inflammation. Ox-OO diets altered ARA metabolism by reducing PPARγ agonists 15d-PGJ2 and 1a,1b-DiHOME-15-deoxy-PGJ2 [29], consistent with reduced PPARγ expression. PGE2, an IL-10 inducer [29], was significantly lower in the ox-OO groups (Fig. S3F), aligning with reduced IL-10 expression (Fig. S1G). Moreover, sEH-derived ARA oxylipin 11,12,15-TriHETrE (Fig. S3I), sEH-derived LA oxylipins 9,10-DiHOME and 12,13-DiHOME (Fig. S3J), and sEH-derived ALA oxylipin 15,16-DiHODE (Fig. S3K) were highest in the HFD-ox-OO group, correlating with increased sEH activity (Fig. 4C–E) and inflammatory response (Fig. 2, Fig. 3), which may explain the inflammation in the duodenum [30]. LOX-derived anti-inflammatory EPA oxylipins, 11-HEPE and 15-oxo-EDE (Fig. S3M), were significantly lower in the ox-OO groups. Most LOX-derived DHA oxylipins, which are PPARγ agonists, were detected exclusively in the HFD group (Fig. S4A), except of 8-HDHA (Fig. S4B), suggesting olive oil diet may affect DHA metabolism. Palmitic and palmitoleic acids were highest in NFD-ox-OO and undetectable in HFD, possibly due to enhanced absorption of free fatty acids in the NFD-ox-OO group (Figs. S4C–D).

In the proximal jejunum, diet significantly influenced oxylipin profiles (Fig. 4A, Figs. S5A–B). Compared to NFD, HFD notably altered DGLA metabolism (Fig. S5C), while ox-OO had no impact on DGLA (Fig. S5C) or adrenic acid (Fig. S5D) metabolism. HFD also enhanced ARA metabolism through COX pathways (Figs. S5E–H) and LOX pathways (Fig. S5I), while ox-OO had minimal effect on ARA metabolism. HFD led to a marked reduction in LA levels (Fig. S5J) due to the lower LA content in lard. Different diets affected the LOX-derived anti-inflammatory metabolite 13-HOTrE, with both HFDs reducing 13-HOTrE levels compared to NFD, and the lowest levels observed in HFD-ox-OO, consistent with PPARγ downregulation (Fig. S1H). HFD-ox-OO also significantly suppressed endogenous EPA synthesis (Fig. S6A), leading to lower levels of anti-inflammatory LOX-derived EPA oxylipins 11-HEPE, 12-HEPE, 15-HEPE, and 15-oxo-EDE (Fig. S6B), which may contribute to inflammation in the proximal jejunum. Compared to NFD, NFD-ox-OO reduced the levels of the anti-inflammatory EPA CYP oxylipin 19-HEPE (Fig. S6C). HFD primarily induced inflammation by inhibiting COX-derived anti-inflammatory oxylipins PGD3 and PGE3 from ARA (Fig. S6D). Similar to the duodenum, EPA LOX-derived oxylipins 7-HDHA, 8-HDHA, 16-HDHA, and 17-HDHA were lowest in ox-OO (Fig. S6E), reflecting changes in PPARγ expression (Fig. S1H). Additionally, palmitic acid (Fig. S5F) and palmitoleic acid (Fig. S5G) showed similar effects in the proximal jejunum as in the duodenum. Moreover, the nitrated product of oleic acid, 10-nitrooleate (Fig. S6H), a PPARγ agonist [31], correlates with changes in PPARγ expression (Fig. S1H).

In the distal jejunum, different diets did not significantly impact the overall oxylipin profiles (Fig. S7A). However, the COX-derived adrenic acid oxylipin dihomo-PGE2 showed the highest concentration in HFD-ox-OO group (Fig. S7C). COX2 expression was also highest in the HFD-ox-OO group (Fig. S7D). Additionally, as a PPARγ agonist, dihomo-PGE2 elevated PPARγ gene expression (Fig. S1I), which may explain the anti-inflammatory effects of ox-OO in the distal jejunum (Fig. 2, Fig. 3).

In the ileum, diets significantly affected the oxylipin profile (Figs. S8A–B), particularly with HFD (Fig. S8A). HFD notably impacted the metabolism of DGLA via COX (Fig. S7C) and LOX (Fig. S8D) pathways, and also inhibits the endogenous synthesis of adrenic acid (Fig. S8E) and promoting COX-derived ARA prostanoids (Figs. S8F–G). In ox-OO groups, COX-derived ARA prostanoids were lower, consistent with COX-2 expression (Fig. S8H), while elevated PGD2 and 11-dehydro-TXB2 in HFD-ox-OO might result from non-enzymatic reactions, potentially contributing to inflammation (Fig. S8I). HFD also enhanced ARA LOX (Fig. S9A) and CYP metabolism (Fig. S9B). Notably, 15-LOX-derived proinflammatory ARA oxylipin 15-oxoETE is upregulated in both ox-OO groups, which could exacerbate inflammation. Both HFD groups suppressed ARA CYP metabolism, as anti-inflammatory 11,12-EET and 14,15-EET levels were significantly lower than in NFD. LA and COX-derived LA oxylipin 13-oxoODE levels were reduced in HFD (Fig. S9E), reflecting lower LA content in HFD. Similarly, ALA and LOX-derived ALA oxylipins were lower in both HFD and NFD-ox-OO groups (Fig. S9F). In HFD, CYP-derived LA oxylipins (9,10-EpOME and 12,13-EpOME; Fig. S9G) and ALA oxylipins (12,13-EpODE and 15,16-EpODE; Fig. S9H) are reduced, as are their sEH-hydrolyzed diols (12,13-DiHOME, 12,13-DiHODE, and 15,16-DiHODE; Figs. S9H–J). In HFD-ox-OO, LA and ALA-derived epoxides and their diols were markedly elevated, potentially contributing to inflammation. Although sEH expression was highest in HFD with low diol levels, HFD-ox-OO showed unchanged sEH expression but increased diol levels (Fig. 4C–E, Fig. S9K). Notably, sEH expression was highest in the HFD group, yet diol levels remained low, whereas in HFD-ox-OO, sEH expression was unchanged, but diol levels were increased. 10-nitrooleate, an sEH inhibitor, was <LOD in HFD but might suppress sEH in other groups (Fig. S9L), suggesting the oxidative conditions in HFD-ox-OO enhanced epoxide-to-diol conversion via non-enzymatic hydrolysis pathways. Additionally, HFD significantly altered EPA and DHA metabolism (Fig. S10 A-D) and increased palmitic acid (Fig. S10E) and palmitoleic acid (Fig. S10F) levels.

In the cecum, diet significantly impacted oxylipin profiles (Figs. S11A–B). HFD influenced the COX metabolism of DGLA (Fig. S11C) and endogenous synthesis of adrenic acid (Fig. S11E), as well as the metabolism of EPA (Fig. S12N) and DHA (Fig. S13). Ox-OO promoted LOX-mediated metabolism of DGLA (Fig. S11D), increasing 15-HETrE concentrations. Compared to NFD, COX-derived dihomo-PGE2 was significantly reduced in HFD, NFD-ox-OO, and HFD-ox-OO, potentially linked to inflammation (Fig. S11E). Similar to the small intestine, HFD affected the COX2-mediated metabolism of ARA (Figs. S11F–H). Ox-OO, however, upregulated pro-inflammatory COX-derived ARA oxylipins PGD2, PGK2, 12-HHTrE, and TXB2 (Fig. S11H), whereas HFD decreased them. This mismatch with COX2 expression suggests altered enzyme activity due to high-fat intake or changes in non-enzymatic reactions caused by dietary oxidized lipids. Additionally, HFD suppressed LOX-mediated metabolism in the cecum (Figs. S12A and C), while ox-OO increased LOX products, especially 5-oxoETE, 11-HETE and 12-HETE, as well as CYP-derived ARA oxylipins were more abundant in the ox-OO groups (Fig. S12D), possibly due to enhanced endogenous ARA synthesis and exogenous intake (Fig. S12B). Furthermore, similar trends were observed for ALA (Fig. S12I) and its oxylipins (Figs. S12J–K), especially CYP-derived 12,13-EpODE and 15,16-EpODE (Fig. S12K). Interestingly, despite higher sEH expression in the cecum compared to other intestinal regions (Fig. 3C–E), its products diols, were undetectable, or in a very low concentration (Fig. S12H), possibly due to the prior hyrolysis of other epoxides, possibly oleic acid-derived 9,10-epoxy-stearic acid (Fig. 43B), as sEH expression in NFD-ox-OO was significantly higher than in NFD (Fig. 3E). Anti-inflammatory LOX-derived oxylipiins 8-HDHA, 11-HDHA and 17-HDHA, and CYP-derived DHA oxylipins 13,14-EpDPE, 16,17-EpDPE and 19,20-EpDPE were significantly higher in ox-OO groups (Fig. S13B), and palmitoleic acid is highest in NFD-ox-OO, but the intake of more oxidized lipids from olive oil might have limit its synthesis (Fig. S13C). Overall, although ox-OO did not trigger severe inflammatory responses in the cecum due to the synergistic effects of anti-inflammatory mediators and the cessation of pro-inflammatory activity, lipid metabolism was affected.

In the colon, diet significantly impacted oxylipin profiles (Figs. S14A–B), with HFD, NFD-ox-OO, and HFD-ox-OO showing similar effects (Fig. S14A). This is evident in the increased COX-mediated metabolism of DGLA (Fig. S14C) and adrenic acid (Fig. S14D), as well as the enhanced COX (Figs. S14E–G) and LOX pathways involving ARA (Fig. S14H). which could contribute to inflammation in the colon. Additionally, the lack of LA in HFD and olive oil diets was reflected in lower levels of LA and its LOX-mediated 13-HODE in the colon (Fig. S15A). Conversely, NFD-ox-OO promoted the CYP-epoxidized 9,10-EpOME and 12,13-EpOME (Fig. S15B), and a similar effect was observed in HFD-ox-OO compared to HFD. Interestingly, sEH-derived 9,10-DiHOME was undetectable, and 12,13-DiHOME levels are extremely low (Fig. S15C). This discrepancy with the high sEH expression in the colon (Fig. 4C–E, Fig. S15D) and the presence of 10-nitrooleate (Fig. S15E), an known sEH inhibitor, suggests that epoxides might be converted into other inflammatory lipid mediators through non-enzymatic reactions involving oxidized lipids in olive oil before they can be converted into diols. Additionally, compared to NFD, ALA, and ALA-derived oxylipins via LOX pathways were significantly reduced in HFD, NFD-ox-OO, and HFD-ox-OO (Fig. S15F). Compared to NFD, NFD-ox-OO decreased levels of EPA (Fig. S15G) and DHA along with their LOX-derived oxylipins (Fig. S15H), as well as reduced levels of the anti-inflammatory palmitoleic acid, contributing to a pro-inflammatory state.

### 3.5 Effects of oxidized olive oil on mitochondrial β-oxidation in intestines

**Fig. 5:**
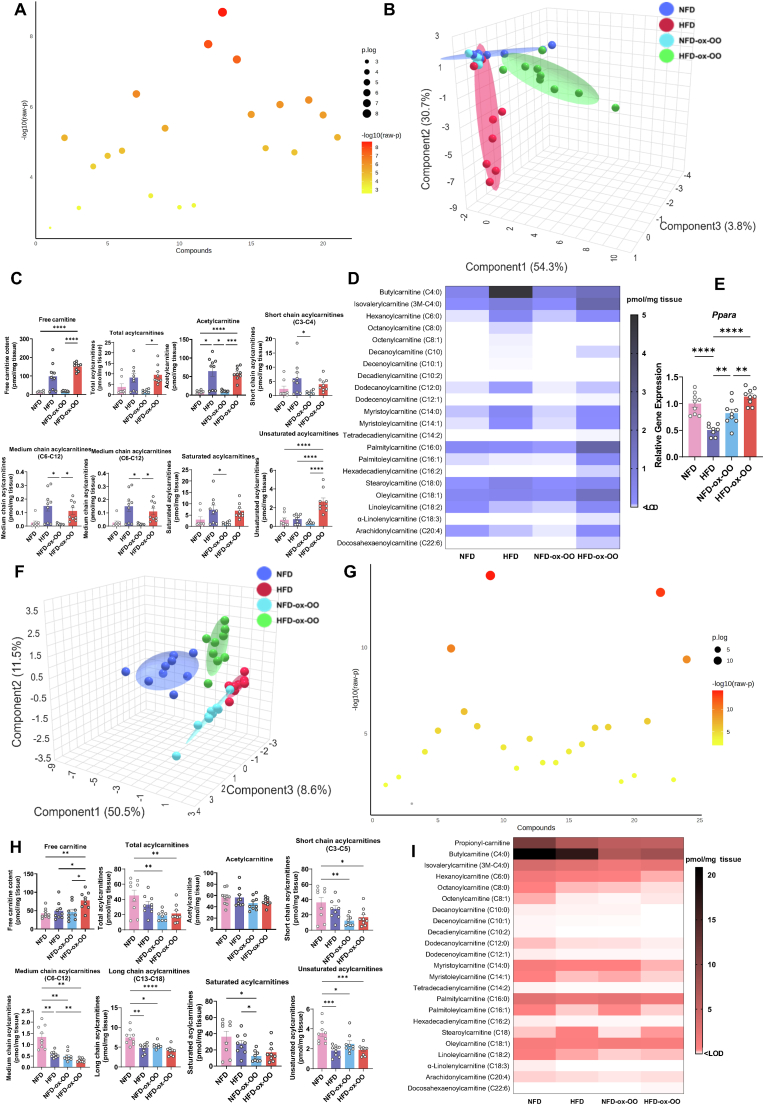
Effects of oxidized olive oil on mitochondrial β-oxidation in intestine. (A) sPLS-DA plot showing the distribution of carnitines in distal jejunum. (B) Statistical differences in carnitine concentrations in distal jejunum among different diets. (C) Concentrations of total acylcarnitines, free carnitine, acetylcarnitine, short chain acylcarnitines, medium chain acylcarnitines, long chain acylcarnitines, saturated acylcarnitines in distal jejunum. (D) Profile of carnitines in distal jejunum. (E) Relative gene expression of PPARα in distal jejunum. (F) sPLS-DA plot showing the distribution of carnitines in colon. (G) Statistical differences in carnitine concentrations in colon among different diets. (H) Concentrations of total acylcarnitines, free carnitine, acetylcarnitine, short chain acylcarnitines, medium chain acylcarnitines, long chain acylcarnitines, saturated acylcarnitines in colon. (I) Profile of carnitines in colon. Data are presented as mean or mean ± SEM. Statistical significance was determined by ANOVA followed by Tukey's post hoc test, ∗p < 0.05, ∗∗p < 0.01, ∗∗∗p < 0.001, ∗∗∗∗p < 0.0001.

Oxylipin metabolism is controlled by mitochondrial β-oxidation during bacterial inflammation, affecting the inflammatory response [11]. To explore the effect of ox-OO on fatty acid metabolism, carnitine profiles and gene expression of CPT1a, LCAD, MCAD, SCAD in the non-inflamed distal jejunum and inflamed ileum, as well as carnitine profiles in the colon were examined (Fig. 5, Figs. S16–18). Different diets significantly impacted the carnitine profile in the distal jejunum (A), with higher levels in HFD and HFD-ox-OO than in NFD and NFD-ox-OO (B-D), as HFD increased mitochondrial fatty acid β-oxidation in the small intestine, enhancing fatty acid uptake. PPARα expression was significantly higher in NFD-ox-OO and HFD-ox-OO compared to HFD (Fig. 5E), further demonstrating the fatty acid β-oxidation-stimulating and anti-inflammatory effects of ox-OO. HFD-ox-OO also increased unsaturated acylcarnitine levels (Fig. 4C), especially linoleylcarnitine, arachidonylcarnitine (Fig. S16P, Fig. S16R), which suggests mitochondrial β-oxidation rather than being converted to proinflammatory oxylipins, thereby reducing inflammation (Figs. 1E and 3).

In the ileum, both ox-OO groups significantly increased mitochondrial β-oxidation, shown by elevated PPARα, CPT1a, SCAD, MCAD, and LCAD expression (Fig. S17A), even under high-fat diet conditions, HFD-ox-OO exhibited stronger mitochondrial β-oxidation than HFD, suggesting that oxidized lipids drive this effect. Comparing carnitine profiles between HFD and HFD-ox-OO distinct differences were observed (Figs. S17C–D), attributed to lower myristoylcarnitine (C14:0) and stearoylcarnitine (C18:0) levels and higher oleoylcarnitine (C18:1) (Figs. S17E–F). Despite stronger mitochondrial β-oxidation, HFD-ox-OO caused more inflammation than HFD, suggesting increased uptake of oleic or oxidized oleic acid, possibly due to non-enzymatic generated diols in the ileum (Fig. S11).

In the colon, except for free carnitine, acylcarnitine levels are significantly reduced in both ox-OO groups, indicating limited fatty acid β-oxidation (Fig. 4H–I, Fig. S18), which is associated with inflammation [11]. Notably, short-chain acylcarnitines, especially propionylcarnitine and butylcarnitine (Fig. 5G, Figs. S18A–B), were significantly reduced, suggesting decreased β-oxidation of butyrate. This further supports inflammation in the colon, highlighting the impact on butyrate production by beneficial colonic microbiota [32].

### 3.6 Effects of oxidized olive oil on gut microbiota composition

**Fig. 6:**
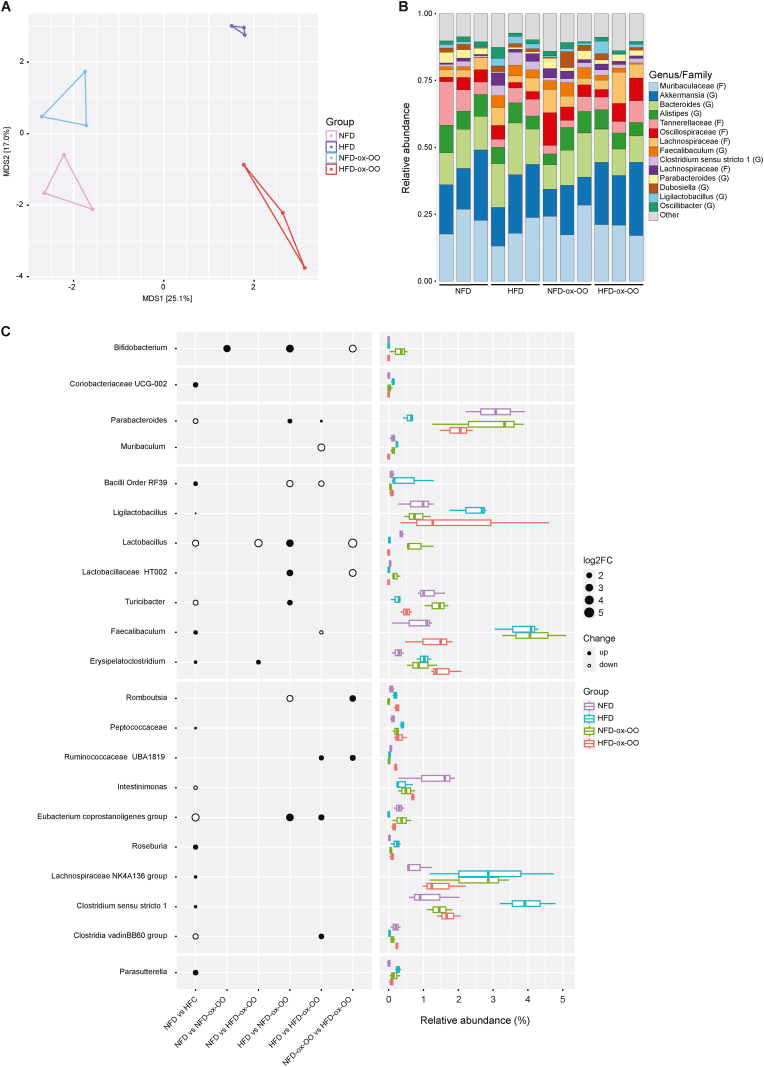
Effects of oxidized olive oil on gut microbiota composition. (A) beta dispersion ANOVA analysis. (B) The composition of colonic microbiota at genius level. (C) Comparison of log2 fold changes in the relative abundance of colonic microbiota between groups.

Ox–OO–induced inflammation (Fig. 2, Fig. 3) and lipid metabolic dysfunction (Fig. 4, Fig. 5) in the colon, especially SCFA metabolism, potentially can be attributed to microbial alterations. Hence, to further investigate the impact of ox-OO on gut microbiome, we analyzed the colonic microbial composition. While Chao1 and Shannon alpha diversity indices showed no significant changes in microbiota abundance (Fig. S19A), beta dispersion ANOVA revealed notable differences among groups (Fig. 6A). Taxonomic analysis showed no major structural changes at the phylum or class levels (Fig.s S19B–C), with minor alterations at the order and family levels (Fig.s S19D-E). Significant compositional changes were evident at the genus level (Fig. 6B).

At the order level, significant changes were observed in the relative abundance of *Clostridia vadinBB60* and *RF39*, although their overall proportions remained relatively low (Fig. 6C). The HFD group showed a higher relative abundance of *RF39* (log2FC = 2) and a lower abundance of *Clostridia vadinBB60*, a butyrate-producing probiotic (log2FC = 2), compared to NFD. The ox-NFD-OO group did not show significant differences in *RF39* and *Clostridia vadinBB60* relative abundances compared to NFD, yet there was a notable decrease in short-chain acylcarnitine levels (Fig. 5H, Figs. S18A–B), suggesting ox-OO affected other SCFA-producing bacteria. At the family level, *Peptococcaceae*, a butyrate-producing bacteria, was significantly more abundant in the HFD group compared to NFD (Fig. 6C), but ox-OO did not alter its abundance. This, along with butylcarnitine level discrepancies, implies ox-OO might influence butyrate production via other bacterial species. Notably, the *Eubacterium coprostanoligenes group*, which produces butyrate [33] and to enhance the intestinal mucus barrier [34], showed reduced abundance in both the HFD and HFD-ox-OO groups compared to NFD and NFD-ox-OO groups, respectively (Fig. 6C). This reduction correlates with decreased butylcarnitine levels (Fig. S18A) and increased inflammation (Fig. 2, Fig. 3, Fig. 4) in the HFD-ox-OO group. Additionally, *Eubacterium coprostanoligenes* is also associated with cholesterol metabolism [35], and its decreased abundance may elevate cholesterol absorption into the bloodstream, potentially leading to lipid metabolism disorders.

At the genus level, diets significantly altered in gut microbiota composition (Fig. 6B). *Coriobacteriaceae UCG-002* exhibited the lowest abundance in the HFD group, whereas *Muribaculum*, *Ligilactobacillus*, *Roseburia*, *Lachnospiraceae NK4A136*, *Clostridium sensu stricto 1*, and *Parasutterella* were most abundant (Fig. 6C). These changes can be attributed to the lipid metabolic disturbances induced by a high-fat diet [36]. Although ox-OO did not significantly alter the overall abundance of these bacterial taxa, *Romboutsia*, associated with obesity and lipid metabolism disorders [37], increased in ox–OO–fed groups, similar to trends between HFD and NFD. SCFA-producing bacteria, such as *Bifidobacterium*, *Lactobacillus*, and *Faecalibaculum* [38], along with dietary lipid metabolism-regulating beneficial bacteria *Parabacteroides* [39] and *Turicibacter* [40], as well as *HT002* [41], were most abundant in the NFD-ox-OO group but reduced in the HFD-ox-OO group (Fig. 6C). The down-regulating of SCFA-producing probiotics can be attributed to the decreased short-chain carnitines in the colon, while the imbalance of lipid-metabolism-regulating microbiota might be associated with both lipid metabolic dysfunction and inflammation. Additionally, the effects of ox-OO on gut microbiota appeared to be dose-dependent: the NFD-ox-OO group up-regulated the abundance of probiotics, whereas the HFD-ox-OO group increased bacteria linked to lipid metabolism disruption and inflammation.

### 3.7 Effects of oxidized olive oil on inflammation response and lipid metabolism in liver

**Fig. 7:**
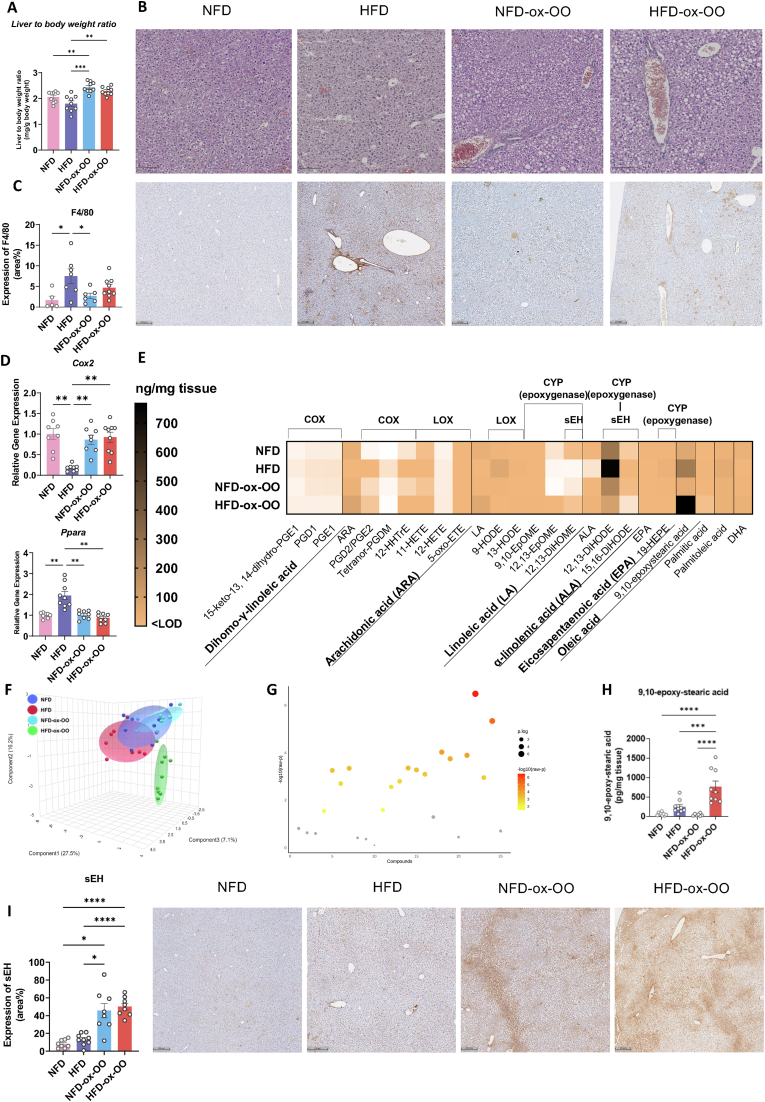
The effects of ox-OO on inflammatory response and lipid metabolism in liver. (A) Liver to body weight ratio. (B) Representative H&E stained liver section. (C) Expression of F4/80 in liver. (D) Relative gene expression of PPARα and COX2 in the liver. (E) Oxylipin profile in liver. (F) sPLS-DA plot showing the distribution of hepatic oxylipins. (G) Statistical differences in hepatic oxylipin concentrations among different diets. (H) 9,10-epoxystearic in the liver (I) Expression of sEH in liver. Data are presented as mean or mean ± SEM. Statistical significance was determined by ANOVA followed by Tukey's post hoc test, ∗p < 0.05, ∗∗p < 0.01, ∗∗∗p < 0.001, ∗∗∗∗p < 0.0001.

As Ox–OO–induced gut microbiota composition alteration potentially impacting hepatic inflammation and lipid metabolism via the gut-liver axis, the impact of ox-OO on liver inflammation and lipid metabolism was investigated. Liver weight-to-body weight ratios were significantly higher in ox-OO groups (Fig. 7A), potentially due to oxidized lipids in ox-OO. In the NFD group, liver tissue appeared healthy, with well-preserved architecture, normal hepatocyte morphology, and no steatosis, inflammation, or fibrosis (Fig. 7B). In contrast, the HFD, NFD-ox-OO, and HFD-ox-OO groups showed signs of hepatocyte ballooning, increased lipid accumulation, and inflammation (Fig. 7B). Both NFD-ox-OO and HFD-ox-OO groups exhibited mild pathological changes, including hepatocyte swelling, lipid droplet accumulation, and fibrosis, indicating progression towards liver injury. The NFD-ox-OO group exhibited moderate steatosis, notable lipid droplet accumulation, early signs of inflammatory cell infiltration, and irregular hepatocyte arrangement with cellular swelling, periportal fibrosis, central vein expansion and deformation, suggesting oxidative stress-related liver damage. The HFD-ox-OO group showed more severe steatosis, marked lipid droplet accumulation, hepatocyte degeneration, periportal fibrosis, and further central vein expansion and deformation. Although F4/80 expression (Fig. 7C) indicated that oxidized olive oil did not significantly increase pro-inflammatory effects, a clear increase in F4/80 expression was observed near the central vein in the HFD-ox-OO group compared to the NFD groups.

Additionally, gene expression of PPARα in the liver was significantly suppressed, while COX2 gene expression was markedly elevated (Fig. 7D), further supporting the presence of an inflammatory trend and lipid metabolism dysfunction, especially in ARA metabolism. These effects on lipid metabolism also extended to other lipid-regulating sites, notably white adipose tissue, where COX2 expression was highest in the HFD-ox-OO group (Fig. S20A). In the NFD and NFD-ox-OO groups, adipocytes were normal in size and morphology, with minimal extracellular matrix deposition. However, the HFD-ox-OO group showed significant adipocyte hypertrophy, irregular distribution, increased inflammatory cell infiltration, and fibrosis, reflecting tissue damage and lipid metabolic dysfunction (Fig. S20B).

We, therefore, hypothesized that oxidized lipids from ox-OO might disrupt liver lipid metabolism, leading to the production of lipid mediators that exert a mild pro-inflammatory effect. This can also be evident from the regulation of oxylipins in the liver, with HFD-ox-OO showing notable differences from the other groups (Fig. 7E–G). COX-derived ARA oxylipins, PGD2/PGE2 and 12-HHTrE (Fig. S20C) were significantly elevated in the HFD and HFD-ox-OO group compared to the NFD group. HFD-ox-OO also upregulated LOX-derived LA oxylipin 13-HODE compared to NFD, contributing to inflammation (Fig. S20D). Notably, CYP (epoxygenase)-regulated LA oxylipins, 9,10-EpOME and 12,13-EpOME, were highest in the HFD-ox-OO group (Fig. S20E), with sEH expression significantly elevated in the ox-OO groups, surpassing even the HFD groups (Fig. 6I). However, ALA-derived sEH diols, 9,10-DiHOME (<LOD), and 12,13-DiHOME levels did not show significant changes (Fig. S20F). Additionally, ALA-derived sEH diols 12,13-DiHODE and 15,16-DiHODE decreased in the ox-OO group compared to HFD (Fig. S20G). These findings contrast with sEH expression (Fig. 7I), suggesting that other epoxides might be metabolized by sEH, potentially inhibiting the metabolism of CYP-derived oxylipins while causing sEH upregulation. Dietary oxidized lipids derived from oleic acid may be converted into other pro-inflammatory mediators through non-enzymatic reactions, such as 9,10-epoxy-stearic acid. Notably, 9,10-epoxy-stearic acid, which had the highest concentration in the HFD-ox-OO group (Fig. 7H), could originate from endogenous epoxidation of oleic acid or dietary intake of ox-OO.

## 4 Discussion

The Mediterranean dietary pattern is suggested as healthy regimen, which uses olive oil as the main source of dietary lipids, which is also rich in oleic acid, with the recommended intake of 30–50 mL for a Mediterranean citizen [42]. Extensive studies have suggested moderate intake of olive oil contributes to health benefits, by reducing the risk of chronic diseases [43]. However, this trend may encourage consumers to excessive intake or over processing of olive oil. While epidemiological studies have suggested that populations in Southern Europe, where olive oil consumption is prevalent, experience lower rates of certain chronic inflammatory conditions [44]. The incidence of inflammatory bowel diseases in some region of Mediterranean coutries has been demonstrated to be increasing [45,46]. This increase suggests that improper consumption or excessive processing of olive oil might contribute to this trend. Our study provides preliminary evidence that oxidized olive oil could have adverse health effects.

**Fig. 8:**
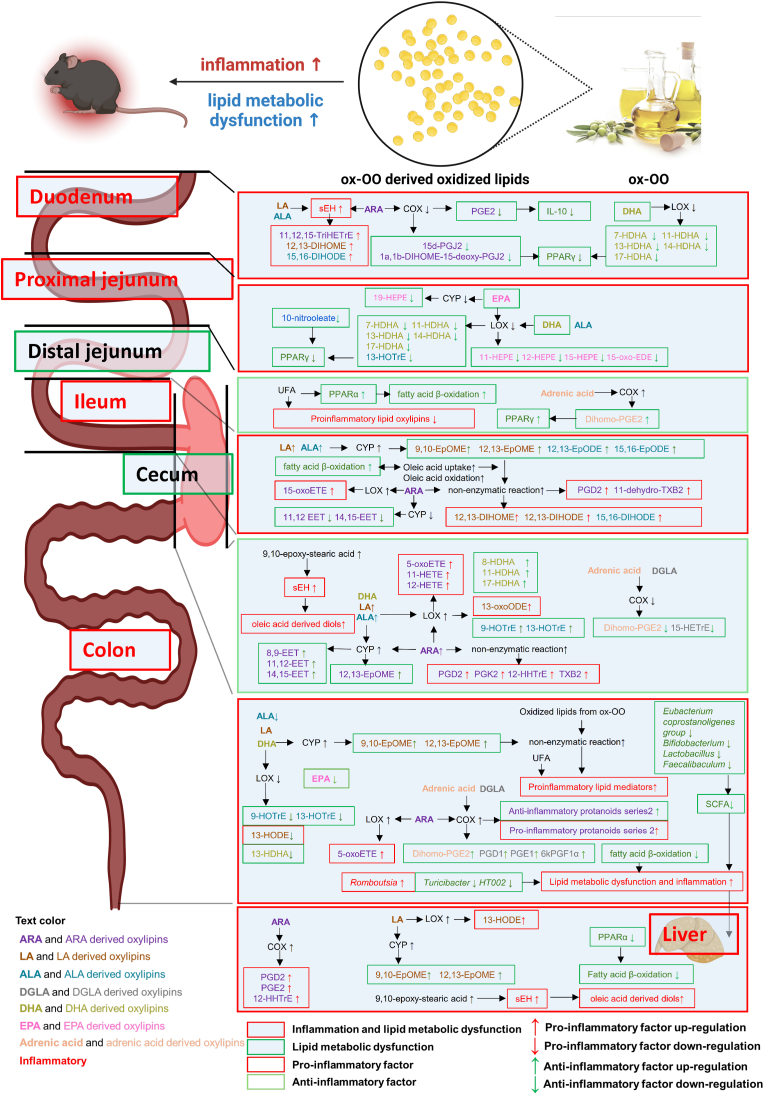
Proposed mechanisms of ox-OO inducing inflammation and lipid metabolic dysfunction.

Our study investigated how ox-OO impacts intestinal and hepatic β-oxidation and fatty acid metabolism, gut microbiota composition, revealing key mechanisms of ox-OO influencing lipid metabolic dysregulation and inflammatory responses (Fig. 8).

In the duodenum, ox-OO altered oxylipin profiles of LA, ALA, ARA, and DHA, leading to a reduction in anti-inflammatory mediators and an increase in pro-inflammatory oxylipins (Fig. 4A, Figs. S3–S4), which induced inflammation (Figs. 2B and 3, Fig. S1A, Fig. S1G). In the proximal jejunum, ox-OO decreased anti-inflammatory EPA-derived oxylipins (and affected EPA, DHA, and ALA profiles (Figs. S17–18), resulting in inflammation (Figs. 2C and 3, Fig. S1B). Conversely, in the distal jejunum, despite high levels of dietary oxidized lipids and a high-fat diet, inflammation was not induced (Figs. 2D and 3, Fig. S1C, Fig. S1I). This may reflect a compensatory mechanism involving upregulation of mitochondrial fatty acid β-oxidation (Fig. 5A–E, Fig. S16), inhibiting PUFA oxygenation and reducing pro-inflammatory oxylipin production (Fig. 4A, Figs. S5–6). In the ileum, despite enhanced mitochondrial β-oxidation (Fig. S17), HFD-ox-OO led to inflammation (Fig. 2E, Fig. S1D, Fig. S1J). This could be attributed to the increased oleoylcarnitine (Fig. S17), suggesting higher oleic acid absorption and potential non-enzymatic oxidation promoting inflammation. In the cecum, changes in pro-/anti-inflammatory oxylipins were observed (Fig. 4A, Figs. S11–13), but these did not trigger significant inflammation (Fig. 2F). However, ox-OO stimulated endogenous synthesis of ARA (Fig. S12B), LA (Fig. S12E), and ALA (Fig. S12I), indicating an impact on lipid metabolism. In the colon, HFD-ox-OO affected the metabolism of multiple fatty acids, resulting in both upregulation and downregulation of pro-/anti-inflammatory oxylipins (Fig. 4A, Figs. S14–15). This imbalance may contribute to severe inflammation and lipid metabolic dysfunction, exacerbated by reduced SCFA-producing probiotics (*Eubacterium coprostanoligenes group*, *Bifidobacterium*, *Lactobacillus*, *Faecalibaculum*) and regulated bacteria associated with lipid dysregulation (*Romboutsia*, *Turicibacter*, *HT002*) (Fig. 5C). This dysfunction extended to the liver, where alterations in ARA and LA metabolism led to changes in pro-/anti-inflammatory oxylipins (Fig. 7, Fig. S20), causing liver lipid metabolic dysfunction disturbances and mild pathology (Fig. 7B).

Overall, ox-OO altered gut and liver lipid metabolism. Specifically, HFD-ox-OO may disrupt lipid metabolism and inflammation in the gut and liver. Interestingly, while PUFA-derived epoxides are known to regulate sEH [15], our findings suggest that 9,10-epoxy-stearic acid from dietary oleic acid may also upregulate sEH in cecum (Fig. 4B–E, Fig. S12M) and liver (Fig. 7H and I). Additionally, oleic acid-derived oxidation products could modulate inflammation through enzymatic or non-enzymatic reactions, a topic requires further investigation.

Our study provides valuable insights into the potential effects of oxidized olive oil on lipid metabolism and inflammation, offering guidance for its healthy use. Epidemiological studies, particularly in regions with high olive oil consumption, are needed to contextualize these effects. Moreover, the effects of 9,10-epoxy-stearic acid and its regulation of sEH activity require further investigation at the cellular and molecular level. Additionally, identifying oleic acid-derived oxidation products formed during food processing or digestion, and assessing their health implications remain critical areas for future research.

## 5 Conclusion

Our findings reveal that oxidized olive oil disrupts lipid metabolism and induces inflammation in both the gut and liver. Oxidized olive oil induced alterations in oxylipin profiles, mitochondrial β-oxidation, and gut microbiota composition. Notably, 9,10-epoxy-stearic acid, was identified as a novel inflammatory dietary lipid mediator that upregulates sEH, highlights the significance of redox biological effects of dietary oxidized lipids. Our results challenge the long-standing notion that olive oil is inherently healthy, indicating that using it as a cooking fat, especially for high-fat foods, may not always be beneficial. To ensure stable essential fatty acid intake and synthesis, combining olive oil with other oils, and taking precautions when using it at higher temperatures may help maintain its health benefits and prevent adverse effects.

## CRediT authorship contribution statement

**Yifan Bao:** Writing – original draft, Visualization, Project administration, Methodology. **Magdalena Osowiecka:** Writing – review & editing, Validation, Methodology, Investigation. **Christiane Ott:** Software, Resources, Investigation, Formal analysis, Data curation. **Vasiliki Tziraki:** Investigation, Formal analysis, Data curation. **Lukas Meusburger:** Investigation, Formal analysis, Data curation. **Claudia Blaßnig:** Investigation, Formal analysis, Data curation. **Daniela Krivda:** Investigation, Formal analysis, Data curation. **Petra Pjevac:** Software, Resources, Investigation, Formal analysis, Data curation. **Joana Séneca:** Software, Resources, Investigation, Formal analysis, Data curation. **Matthias Strauss:** Validation, Formal analysis. **Christina Steffen:** Investigation, Formal analysis, Data curation. **Verena Heck:** Investigation, Formal analysis, Data curation. **Soner Aygün:** Investigation, Formal analysis, Data curation. **Kalina Duszka:** Software, Resources, Investigation, Formal analysis, Data curation. **Kevin Doppelmayer:** Investigation, Formal analysis, Data curation. **Tilman Grune:** Software, Resources, Investigation, Formal analysis, Data curation. **Marc Pignitter:** Writing – review & editing, Supervision, Funding acquisition, Conceptualization.

## Declaration of competing interest

The authors declare no competing interests.

## Data Availability

Data will be made available on request.
