# Supplementary material for: Dietary oxidized lipids in redox biology: Oxidized olive oil disrupts lipid metabolism and induces intestinal and hepatic inflammation in C57BL/6J mice
Source: Redox Biol. 2025 Mar 1;81:103575. doi: 10.1016/j.redox.2025.103575 (PMC11927754; doi:10.1016/j.redox.2025.103575)
Supplement: Multimedia component 1 [file mmc1.docx]

**Table S1** Primary antibodies incubation

|  | Anti-sEH Antibody  (Santa Cruz Biotechnology, #sc-166961) | | Anti-F4/80 antibody  (Abcam, #ab300421) | |
| --- | --- | --- | --- | --- |
|  | Dillution | Incubation time | Dilution | Incubation time |
| Duodenum | 1:20 | 12-24 h | 1:50 | 12-24 h |
| Proximal jejunum | 1:20 | 12-24 h | 1:10 | 36-48 h |
| Distal jejunum | 1:20 | 12-24 h | 1:20 | 36-48 h |
| Ileum | 1:10 | 12-24 h | 1:150 | 12-24 h |
| Cecum | 1:20 | 12-24 h | 1:50 | 12-24 h |
| Colon | 1:10 | 12-24 h | 1:150 | 12-24 h |
| Liver | 1:50 | 12-24 h | 1:100 | 12-24 h |

**Table S2** Primer sequences of the genes

| **Gene** | **Direction** | **Sequence** |
| --- | --- | --- |
| *Cpt1a* | FW | 5’-GCCCTGAGACAGACTCACAC-3’ |
|  | RV | 5’-GTCCATTTTCCTTCCGTGCG-3’ |
| *LCAD* | FW | 5’-CTACCTCATGCAAGAGCTTCCACA-3’ |
|  | RV | 5’-CTTCAAACATGAACTCACAGGCAGA-3’ |
| *MCAD* | FW | 5’-TGATGTGGCGGCCATTAAGA-3’ |
|  | RV | 5’-GGGTTAGAACGTGCCAACAAGAA-3’ |
| *SCAD* | FW | 5’-TGGCGACGGTTACACACTG-3’ |
|  | RV | 5’-GTAGGCCAGGTAATCCAAGCC-3’ |
| *TNF-α* | FW | 5’-CCACAGGGCTGCAATTTTCC-3’ |
|  | RV | 5’-CCACAGGGCTGCAATTTTCC-3’ |
| *IL-1β* | FW | 5’-TCCTGTGTAATGAAAGACGGC-3’ |
|  | RV | 5’-GGTGCTGATGTACCAGTTGGG-3’ |
| *IL-10* | FW | 5’-GCTCTTACTGACT66CATGAG-3’ |
|  | RV | 5’-CGCAGCTCTAGGAGCATGTG-3’ |
| *TLR4* | FW | 5`-ATGGCATGGCTTACACCACC-3´ |
|  | RV | 5`-GAGGCCAATTTTGTCTCCACA-3´ |
| *COX-2* | FW | 5`-TGAGCAACTATTCCAAACCAGC-3´ |
|  | RV | 5`-GCACGTAGTCTTCGATCACTATC-3´ |
| *PPARα*  (liver) | FW | 5`-TGGTTGAATCGTGAGGAACA-3´ |
|  | RV | 5`-ATCGCCACTAAGGTGTCAGG-3´ |
| *PPARα*  (intestine and white adipose tissue) | FW | 5`-AGAGCCCCATCTGTCCTCTC-3´ |
|  | RV | 5`-ACTGGTAGTCTGCAAAACCAAA-3´ |
| *PPARγ* | FW | 5`-GTGCCAGTTTCGATCCGTAGA-3´ |
|  | RV | 5`-GGCCAGCATCGTGTAGATGA-3´ |
| *NFkB p65* | FW | 5`-CCAGACACAGATGATCGCCAC-3´ |
|  | RV | 5`-GACAGAAGTTGAGTTTCGGGTAGG-3´ |
| *GAPDH* | FW | 5`-CCTTCATTGACCTCAACTAC-3´ |
|  | RV | 5`-GGAAGGCCATGCCAGTGACC-3´ |
| *18S* | FW | 5`-CGGCTACCACATCCAAGGAA-3´ |
|  | RV | 5`-GCTGGAATTACCGCGGCT-3´ |
| *HPRT1* (WAT) | FW | 5`-CTGGTGAAAAGGACCTCTCG-3´ |
|  | RV | 5`-TGAAGTACTCATTATAGTCAAGGGCA-3´ |

**Table S3** The concentrations and sMRM of oxylipins internal standards

| **Standard** | **Concentration** | **Precursor m/z** | **Product m/z** |
| --- | --- | --- | --- |
| Thromboxane B2-d4 | 0.5 (μg/mL) | 373.3 | 173.2 |
| 9(S)-HODE-d4 | 0.5 (μg/mL) | 299.2 | 172.3 |
| 9(10)-DiHOME-d4 | 0.5 (μg/mL) | 317.2 | 203.4 |
| 5(S)-HETE-d8 | 0.5 (μg/mL) | 327.5 | 116 |
| 9(10)-EpOME-d4 | 0.5 (μg/mL) | 299.2 | 281.2 |
| 12(13)-EpOME-d4 | 0.5 (μg/mL) | 299.1 | 280 |
| PGE2-d4 | 0.5 (μg/mL) | 355.2 | 275.3 |
| 10-Nitrooleate-d17 | 0.5 (μg/mL) | 344.3 | 46.05 |

**Table S4** sMRM transitions of the oxylipins

| **Precursor type** | **Precursor name** | **Precursor m/z** | **Product m/z** |
| --- | --- | --- | --- |
| Epoxide | 11,12-EpETE | 317.2 | 172.3 |
|  | 11,12-EET | 319.3 | 203.4 |
|  | 11,12-EET | 319.3 | 116 |
|  | 12,13-EpODE | 293.2 | 281.2 |
|  | 12,13-EpOME | 295.2 | 280 |
|  | 13,14-EpDPE | 343.2 | 275.3 |
|  | 14,15-EpETE | 317.2 | 46.05 |
|  | 14,15-EET | 319.2 | 175.2 |
|  | 14,15-EET | 319.2 | 219.2 |
|  | 15,16-EpODE | 293.3 | 235.2 |
|  | 16,17-EpDPE | 343.2 | 201 |
|  | 16,17-EpDPE | 343.2 | 233.2 |
|  | 19,20-EpDPE | 343.2 | 241.2 |
|  | 19,20-EpDPE | 343.2 | 241.2 |
|  | 5,6-EET | 319.2 | 191.1 |
|  | 8,9-EET | 319.2 | 151.2 |
|  | 8,9-EET | 319.2 | 155.2 |
|  | 9,10-EpOME | 295.3 | 171.1 |
|  | 9,10-EpOME | 295.3 | 277 |
| Fatty Acid | 10-nitrooleate | 326 | 181 |
|  | Palmitoleic acid | 253 | 209 |
|  | Palmitic acid | 255 | 211 |
|  | EPA | 301 | 257 |
|  | ARA | 303 | 259 |
|  | ALA | 277.25 | 259.15 |
|  | LA | 279.2 | 261.25 |
|  | 10-nitrooleate | 326 | 279 |
|  | DHA | 327 | 283 |
|  | Adrenic Acid | 331 | 287 |
|  | 9(10)-epoxy-stearic acid | 297 | 170.8 |
| Hydroxy Fatty Acid | 10-HDHA | 343 | 153 |
|  | 10-HDHA | 343 | 181 |
|  | 11,12,15-TriHETrE | 353.2 | 167.1 |
|  | 11,12-DiHETrE | 337.2 | 167.1 |
|  | 11,12-DiHETrE | 337.2 | 169 |
|  | 11-HDHA | 343.2 | 121.1 |
|  | 11-HEPE | 317 | 167 |
|  | 11-HEPE | 317 | 195 |
|  | 11-HETE | 319.2 | 167.2 |
|  | 12,13-DiHODE | 311.2 | 183.1 |
|  | 12,13-DiHOME | 313.2 | 129.1 |
|  | 12,13-DiHOME | 313.2 | 183.2 |
|  | 12-HEPE | 317.2 | 179.2 |
|  | 12-HETE | 319.2 | 179.2 |
|  | 12-HHTrE | 279.1 | 179.1 |
|  | 12-HHTrE | 279.1 | 217.1 |
|  | 13-HDHA | 343.2 | 193.1 |
|  | 13-HODE | 295.2 | 113.1 |
|  | 13-HODE | 295.2 | 195.2 |
|  | 13-HOTrE | 293 | 113 |
|  | 13-HOTrE | 293.2 | 195.1 |
|  | 14,15-DiHETrE | 337.2 | 207.1 |
|  | 14-HDHA | 343.2 | 205.2 |
|  | 14-HDHA | 343.2 | 234.2 |
|  | 15,16-DiHODE | 311.2 | 223.2 |
|  | 15-HEPE | 317.2 | 175 |
|  | 15-HEPE | 317.2 | 219.2 |
|  | 15-HETE | 319.2 | 219.2 |
|  | 15-HETrE | 321 | 221 |
|  | 15-HETrE | 321.2 | 221.2 |
|  | 16-HDHA | 343.2 | 189.2 |
|  | 16-HDHA | 343.2 | 233.2 |
|  | 16-HETE | 319 | 189 |
|  | 17-HDHA | 343.2 | 201.2 |
|  | 17-HDHA | 343.2 | 245.2 |
|  | 18-HEPE | 317.2 | 215 |
|  | 18-HEPE | 317.2 | 259.2 |
|  | 19-HEPE | 317.2 | 229.3 |
|  | 19-HETE | 319 | 177 |
|  | 5-HETE | 319.2 | 115.2 |
|  | 7-HDHA | 343.2 | 141.2 |
|  | 7-HDHA | 343.2 | 201.2 |
|  | 8,9-DiHETrE | 337.2 | 127.1 |
|  | 8-HDHA | 343.2 | 109.2 |
|  | 8-HDHA | 343.2 | 189.2 |
|  | 8-HETE | 319.2 | 155.2 |
|  | 8-HETrE | 321 | 163 |
|  | 9,10-DiHOME | 313.2 | 171.1 |
|  | 9,10-DiHOME | 313.2 | 201.2 |
|  | 9-HETE | 319.2 | 167.2 |
|  | 9-HODE | 295.2 | 171.1 |
|  | 9-HOTrE | 293.2 | 171.2 |
|  | 13-oxoODE | 293.2 | 113.1 |
| Keto Fatty Acid | 15-oxoETE | 317.2 | 113.1 |
|  | 15-oxoETE | 317.2 | 139 |
|  | 9-oxoODE | 293.2 | 185.1 |
|  | 15-oxoEDE | 321 | 195 |
|  | 9-oxoODE | 293.2 | 197.1 |
|  | 5-oxoETE | 317.2 | 203.2 |
|  | 15-oxoEDE | 321 | 223 |
|  | 11 beta-PGE2 | 351 | 271 |
| Prostaglandin | 11 beta-PGE2 | 351 | 315 |
|  | 11 beta-PGF2α | 353.3 | 193.1 |
|  | 11 beta-PGF2α | 353.3 | 309.1 |
|  | 15-keto-13,14-dihydro-PGE1 | 353.3 | 221.2 |
|  | 13,14-dihydro-15-keto-PGF2a | 353.3 | 183.3 |
|  | 15 keto-PGF1α | 353 | 193 |
|  | 15-dk,dh-PGF1α | 369 | 223 |
|  | 15d-PGA2 | 315 | 187 |
|  | 15d-PGD2 | 333 | 271 |
|  | 15d-PGD2 | 333 | 315 |
|  | 15d-PGJ2 | 315.2 | 203.2 |
|  | 15d-PGJ2 | 315.2 | 271.2 |
|  | 15k-PGE2 | 349 | 287 |
|  | 15k-PGE2 | 349 | 331 |
|  | 1a, 1b-dihomo-15deoxy-PGJ2 | 343.2 | 233.15 |
|  | 1a, 1b-dihomo-15deoxy-PGJ2 | 343.2 | 299.2 |
|  | 20 OH-PGE2 | 367 | 331 |
|  | 20 OH-PGE2 | 367 | 349 |
|  | 20 OH-PGF2 α | 369 | 325 |
|  | 6k PGF1α | 369 | 163 |
|  | 6k PGF1α | 369 | 245 |
|  | 8-iso PGF2α | 353 | 291 |
|  | 8-iso PGF2α | 353 | 309 |
|  | 8-iso PGF2all | 353 | 309 |
|  | 8-iso PGF3α | 351 | 307 |
|  | bicyclo PGE2 | 333 | 235 |
|  | d17 6k PGF1α | 367 | 163 |
|  | d17 6k PGF1α | 367 | 243 |
|  | dh PGF2α | 355 | 337 |
|  | dhk PGD2/E2 | 351 | 315 |
|  | dhk PGF2α | 353 | 113 |
|  | dhk PGF2α | 353 | 195 |
|  | dihomo PGE2 | 379 | 343 |
|  | dihomo PGE2 | 379 | 361 |
|  | PGA2 | 333 | 271 |
|  | PGA2 | 333 | 315 |
|  | PGB2 | 333.3 | 175.1 |
|  | PGB2 | 333 | 235 |
|  | PGD1 | 353.3 | 317.2 |
|  | PGD2 | 351.2 | 315 |
|  | PGD3 | 349.3 | 269.2 |
|  | PGE1 | 353.3 | 273.2 |
|  | PGE1 | 353.3 | 317.2 |
|  | PGE2 | 351.2 | 271.3 |
|  | PGE2 | 351.2 | 315 |
|  | PGE3 | 349 | 313 |
|  | PGEM | 351 | 315 |
|  | PGEM | 351 | 333 |
|  | PGF1α | 355 | 311 |
|  | PGF2α | 353.2 | 309.2 |
|  | PGF3α | 351 | 307 |
|  | PGFM | 353 | 183 |
|  | PGFM | 353 | 223 |
|  | PGJ2 | 333.3 | 189.2 |
|  | PGJ2 | 333.3 | 233 |
|  | PGK2 | 349 | 249 |
|  | PGK2 | 349 | 287 |
|  | Resolvin D1 | 375.3 | 141 |
| Resolvin | Resolvin D1 | 375.3 | 215 |
|  | Resolvin E1 | 349.3 | 161 |
|  | Resolvin E1 | 349.3 | 195 |
|  | 11-dehydro-TXB2 | 367 | 161 |
| Thromboxane | 11-dehydro-TXB2 | 367 | 305 |
|  | TXB1 | 371.3 | 171.2 |
|  | TXB1 | 371.3 | 197 |
|  | TXB2 | 369.2 | 169.1 |
|  | TXB2 | 369.2 | 195 |

Abbreviations

EpETE: Epoxy-Eicosatetraenoic Acid; EET: Epoxy-Eicosatrienoic Acid; EpODE: Epoxy-Octadecadienoic Acid; EpOME: Epoxy-Octadecamonoenoic Acid; EpDPE: Epoxy-Docosapentaenoic Acid; DHA: Docosahexaenoic Acid; HDHA: Hydroxy-Docosahexaenoic Acid; ALA: Alpha-Linolenic Acid; TriHETrE: Trihydroxy-Eicosatrienoic Acid; DiHETrE: Dihydroxy-Eicosatrienoic Acid; HEPE: Hydroxy-Eicosapentaenoic Acid; DiHODE: Dihydroxy-Octadecadienoic Acid; DiHOME: Dihydroxy-Octadecenoic Acid; HETE: Hydroxy-Eicosatetraenoic Acid; HHTrE: Hydroxy-Eicosatrienoic Acid; HODE: Hydroxy-Octadecadienoic Acid; HOTrE: Hydroxy-Octadecatrienoic Acid; HETrE: Hydroxy-Eicosatrienoic Acid; oxoODE: Oxo-Octadecadienoic Acid; oxoETE: Oxo-Eicosatetraenoic Acid; oxoEDE: Oxo-Eicosadienoic Acid; PGE: Prostaglandin E; PGF: Prostaglandin F; PGD: Prostaglandin D; PGA: Prostaglandin A; TXB: Thromboxane B

**Table S5** The concentrations of internal standards of carnitines and lipids

| **Standard** | **Concentration** |
| --- | --- |
| Acteylcarnitine-d3 (chloride) | 1 µM (jejunum distal, ileum)  75 µM (colon) |
| L-carnitine-d3 (chloride) | 1 µM (jejunum distal, ileum)  30 µM (colon) |
| Myristoylcarnitine-d3 (chloride) | 1 µM (jejunum distal, ileum)  0.17 µM (colon) |
| Oleoylcarnitine-d3 (chloride) | 1.0 µM |

**Table S6** MRM overview of carnitine molecules

| **Molecule Name** |  | **Precursor [m/z]** | **Product [m/z]** |
| --- | --- | --- | --- |
| Free carnitine | | 162.1 | 85.1 |
| Acetylcarnitine (C2) | | 204.1 | 85.1 |
| Propionylcarnitine (C3) | | 218.1 | 85.1 |
| Butylcarnitine (C4) | | 232.2 | 85.1 |
| Isovalerylcarnitine (3M-C4) | | 246.2 | 85.1 |
| Hexanoylcarnitine (C6) | | 260.2 | 85.1 |
| Octanoylcarnitine (C8) | | 288.2 | 85.1 |
| Octenylcarnitine (C8:1) | | 286.2 | 85.1 |
| Decanoylcarnitine (C10) | | 316.2 | 85.1 |
| Decenoylcarnitine (C10:1) | | 314.2 | 85.1 |
| Decadienylcarnitine (C10:2) | | 312.2 | 85.1 |
| Dodecanoylcarnitine (C12) | | 344.3 | 85.1 |
| Dodecenoylcarnitine (C12:1) | | 342.3 | 85.1 |
| Myristoylcarnitine (C14) | | 372.3 | 85.1 |
| Myristoleylcarnitine (C14:1) | | 370.3 | 85.1 |
| Tetradecadienylcarnitine (C14:2) | | 368.3 | 85.1 |
| Palmitylcarnitine (C16) | | 400.3 | 85.1 |
| Palmitoleylcarnitine (C16:1) | | 398.3 | 85.1 |
| Hexadecadienylcarnitine (C16:2) | | 396.3 | 85.1 |
| Stearoylcarnitine (C18) | | 428.4 | 85.1 |
| Oleylcarnitine (C18:1) | | 426.4 | 85.1 |
| Linoleylcarnitine (C18:2) | | 424.3 | 85.1 |
| α-Linoleylcarnitine (C18:3) | | 422.3 | 85.1 |
| Arachidonylcarnitine (C20:4) | | 448.3 | 85.1 |
| Eicosapentenylcarnitine (C20:5) | | 446.3 | 85.1 |
| Docosahexaenoylcarnitine (C22:6) | | 472.3 | 85.1 |
| Acteylcarnitine-d3 | | 165.1 | 85.1 |
| L-carnitine-d3 | | 207.1 | 85.1 |
| Myristoylcarnitine-d3 | | 375.3 | 85.1 |
| Oleoylcarnitine-d3 | | 429.4 | 85.1 |
