# Supplementary material for: Dietary oxidized lipids in redox biology: Oxidized olive oil disrupts lipid metabolism and induces intestinal and hepatic inflammation in C57BL/6J mice
Source: Redox Biol. 2025 Mar 1;81:103575. doi: 10.1016/j.redox.2025.103575 (PMC11927754; doi:10.1016/j.redox.2025.103575)
Supplement: Multimedia component 2 [file mmc2.docx]

**Figure S1** Effects of oxidized olive oil on the area% of F4/80 and gene expression levels of IL-10, TNF-α, IL-1β, NF-kB p65, TLR4 and PPARγ in the intestines. Percent area of F4/80 in the duodenum (A), proximal jejunum (B), distal jejunum (C), ileum (D), and colon (E).(G) Gene expression levels of IL-10 and PPARγ in the duodenum. (H) Gene expression levels of IL-10, TNF-α, PPARγ, and PPARα in the proximal jejunum. (J) Gene expression levels of IL-10, NF-κB p65, TLR4, and PPARγ in the duodenum. (K) Gene expression levels of IL-10 and NF-κB p65 in the cecum. (L) Gene expression levels of IL-10, TNF-α, NF-κB p65, and TLR4 in the colon. Data are presented as mean or mean ± SEM. Statistical significance was determined by ANOVA followed by Tukey's post hoc test. *p < 0.05, **p < 0.01, ***p < 0.001, **** p < 0.0001.

**Figure S2** Effects of oxidized olive oil on oxylipins in the intestines. (A) sPLS-DA plot showing the distribution of oxylipins in the duodenum, proximal jejunum, distal jejunum, ileum, cecum, and colon across the NFD, HFD, NFD-ox-OO, and HFD-ox-OO groups. (B) Statistical differences in oxylipin levels among intestinal regions (C) and different diets (D). Statistical significance was determined by ANOVA followed by Tukey's post hoc test.

**Figure S3** Oxylipin profiles and gene expression in the duodenum. (A) sPLS-DA plot showing the distribution of oxylipins in the duodenum. (B) Statistical differences in oxylipin concentrations among different diets. (C) COX-derived-DGLA oxylipins PGD1 and PGE1. (D) LOX-derived-DGLA oxylipin 15-HETrE. (E) Gene expression level of COX2. (F) COX-derived ARA oxylipins PGE2, 11-β-PGE2, TXB2. (G) COX-derived ARA oxylipins 15d-PGJ2, 1a, 1b-DIHOME-15-deoxy-PGJ2. (H) LOX-derived ARA oxylipins 15-HETE, 15-oxoETE. (I) CYP-sEH-derived ARA oxylipin 11,12,15-TriHETrE. (J) CYP-sEH-derived LA oxylipins 9,10-DIHOME, 12,13-DIHOME. (K) CYP-sEH-derived ALA oxylipin 15,16-DIHODE. (L) LOX-derived EPA oxylipins 11-HEPE and 15-oxoEDE. Statistical significance was determined by ANOVA followed by Tukey's post hoc test. *p < 0.05, **p < 0.01, ***p < 0.001, **** p < 0.0001.

**Figure S4** LOX-Derived DHA oxylipins and key fatty acids in the duodenum. LOX-derived DHA oxylipins 7-HDHA, 11-HDHA, 13-HDHA, 14-HDHA, 17-HDHA (A) and 8-HDHA (B). (C) Palmitic acid. (D) Palmitoleic acid. Statistical significance was determined by ANOVA followed by Tukey's post hoc test. **** p < 0.0001.

**Figure S5** Oxylipin profiles and gene expression in the proximal jejunum. (A) sPLS-DA plot showing the distribution of oxylipins in the proximal jejunum. (B) Statistical differences in oxylipin concentrations among different diets. (C) COX-derived-DGLA oxylipins PGD1, PGE1, 13,14-dihydro-15-keto-PGE1, PGF1α, 6k PGF1α and TXB1. (D) Adrenic acid and COX-derived adrenic acid oxylipin dihomo-PGE2. (E) ARA. (F) Gene expression level of COX2. (G) COX-derived ARA oxylipins PGE2 and PGF2α. (H) COX-derived ARA oxylipins 11-β-PGF2α, dhk PGF2α, 8-iso-PGF2α and TXB2. (I) LOX-derived ARA oxylipins 5-oxo-ETE and 12-HETE. (J) LA. (K) LOX-derived ALA oxylipins 13-HOTrE. Statistical significance was determined by ANOVA followed by Tukey's post hoc test. *p < 0.05, **p < 0.01, ***p < 0.001, **** p < 0.0001.

**Figure S6** EPA and DHA derived oxylipins and key fatty acids in the proximal jejunum. (A) EPA. (B) LOX-derived EPA oxylipins 11-HEPE, 12-HEPE, 15-HEPE, 15-oxoEDE. (C) CYP-derived EPA oxylipin 19-HEPE. (D) COX-derived EPA oxylipins PGD3 and PGE3. (E) LOX-derived DHA oxylipins 7-HDHA, 8-HDHA, 14-HDHA, 16-HDHA, 17-HDHA. (F) Palmitic acid. (G) Palmitoleic acid. (H) 10-nitrooleate. Statistical significance was determined by ANOVA followed by Tukey's post hoc test. *p < 0.05, **p < 0.01, ***p < 0.001, **** p < 0.0001.

**Figure S7** Oxylipin profiles and gene expression in the distal jejunum. (A) sPLS-DA plot showing the distribution of oxylipins in the distal jejunum. (B) Statistical differences in oxylipin concentrations among different diets. (C) COX-derived adrenic acid oxylipin dihomo-PGE2. (D) Gene expression of COX2. Statistical significance was determined by ANOVA followed by Tukey's post hoc test. *p < 0.05, **p < 0.01, ***p < 0.001, **** p < 0.0001.

**Figure S8** Oxylipin profiles and gene expression in the ileum. (A) sPLS-DA plot showing the distribution of oxylipins in the ileum. (B) Statistical differences in oxylipin concentrations among different diets. (C) COX-derived-DGLA oxylipins PGE1, 13,14-dihydro-15-keto-PGE1, 6k PGF1α. (D) LOX-derived-DGLA oxylipin 15-HETrE. (E) Adrenic acid. (F) COX-derived ARA oxylipins PGE2, PGD2/E2, PGA2, 15k-PGE2, 20 OH-PGE2α, 8-iso-PGF2 α, PGJ2, 15d-PGJ2 and 12-HHTrE. (H) Gene expression of COX2. (I) COX-derived ARA oxylipin PGD2 and 11-dehydro-TXB2. Statistical significance was determined by ANOVA followed by Tukey's post hoc test. *p < 0.05, **p < 0.01, ***p < 0.001, **** p < 0.0001.

**Figure S9** ARA, LA and ALA oxylipin profiles and sEH expression in the ileum. (A) LOX-derived ARA oxylipins 9-HETE, 5-HETE, 5-oxo-ETE, 11-HETE and 12-HETE. (B) CYP-derived ARA oxylipins 19-HETE. (C) LOX-derived ARA oxylipin 15-oxoETE. (D) CYP-derived ARA oxylipins 11,12-EET and 14,15-EET. (E) LA and LOX-derived LA oxylipin 13-oxoODE. (F) ALA and LOX-derived ALA oxylipin 9-HOTrE. (G) CYP-derived ARA oxylipin 9,10-EpOME and 12,13-EpOME. (H) sEH-derived ARA oxylipin 9,10-DIHOME and 12,13-DIHOME. (I) CYP-derived ALA oxylipin 12,13-EpODE and 15,16-EpODE. (J) sEH-derived 12,13-DIHODE and 15,16-DIHODE. (K) sEH expression in the ileum. (L) 10-nitrooleate. Statistical significance was determined by ANOVA followed by Tukey's post hoc test. *p < 0.05, **p < 0.01, ***p < 0.001, **** p < 0.0001.

**Figure S10** EPA and DHA derived oxylipins and key fatty acids in the ileum. (A) LOX-derived EPA oxylipins 12-HEPE and 15-oxoEDE. (B) CYP-derived EPA oxylipin 19-HEPE. (C) LOX-derived DHA oxylipins 7-HDHA, 10-HDHA and 13-HDHA. (D) LOX-derived DHA oxylipins 8-HDHA, 11-HDHA, 14-HDHA, 16-HDHA and 17-HDHA. (E) Palmitic acid. (F) Palmitoleic acid. Statistical significance was determined by ANOVA followed by Tukey's post hoc test. *p < 0.05, **p < 0.01, ***p < 0.001, **** p < 0.0001.

**Figure S11** Oxylipin profiles and gene expression in the cecum. (A) sPLS-DA plot showing the distribution of oxylipins in the cecum. (B) Statistical differences in oxylipin concentrations among different diets. (C) COX-derived-DGLA oxylipins PGD1, PGE1, 13,14-dihydro-15-keto-PGE1, PGF1α, 6k PGF1α and 15-keto-PGF1α. (D) LOX-derived-DGLA oxylipin 15-HETrE. (E) Adrenic acid and COX-derived adrenic acid dihomo-PGE2. (F) COX-derived ARA oxylipins dhkPGD2/E2, PGE2 and 11β-PGE2. (G) COX-derived ARA oxylipins 15k-PGE2, PGA2, PGF2α, 11β-PGEα, 15d-PGJ2 and 1a,1b-DIHOME-15-deoxy-PGJ2. (H) COX-derived ARA oxylipins PGD2, PGK2, 12-HHTrE, TXB2. (I) Gene expression of COX2. Statistical significance was determined by ANOVA followed by Tukey's post hoc test. *p < 0.05, **p < 0.01, ***p < 0.001, **** p < 0.0001.

**Figure S12** ARA, LA and ALA oxylipin profiles and sEH expression in the cecum. (A) LOX-derived ARA oxylipins 5-HETE, 5-oxo-ETE, 8-HETE, 11-HETE and 12-HETE. (B) ARA. (C) LOX-derived ARA oxylipins 15-HETE and 15-oxoETE. (D) CYP-derived ARA oxylipins 5,6-EET, 8,9-EET, 11,12-EET and 14,15-EET. (E) LA. (F) LOX-derived LA oxylipin 13-oxoODE. (G) CYP derived LA oxylipin 12,13-EpOME. (H) sEH-derived LA oxylipin 9,10-DIHOME. (I) ALA. (J) LOX-derived ALA oxylipin 9-HOTrE and 13-HOTrE. (K) CYP-derived ALA oxylipin 12,13-EpODE and 15,16-EpODE. (L) EPA, COX-derived EPA oxylipin PGD3, LOX-derived EPA oxylipin 15-oxoEDE. Statistical significance was determined by ANOVA followed by Tukey's post hoc test. *p < 0.05, **p < 0.01, ***p < 0.001, **** p < 0.0001.

**Figure S13** DHA derived oxylipins and palmitoleic acid in the cecum. (A) LOX-derived DHA oxylipins 7-HDHA, 8-HDHA, 11-HDHA, 14-HDHA, 16-HDHA and 17-HDHA. (B) CYP derived EPA oxylipins 13,14-EpDPE, 16,17-EpDPE and 19,20- EpDPE. (C) Palmitic acid. Statistical significance was determined by ANOVA followed by Tukey's post hoc test. *p < 0.05, **p < 0.01, ***p < 0.001, **** p < 0.0001.

**Figure S14** Oxylipin profiles and gene expression in the colon. (A) sPLS-DA plot showing the distribution of oxylipins in the colon. (B) Statistical differences in oxylipin concentrations among different diets. (C) COX-derived-DGLA oxylipins PGD1, PGE1, 6k PGF1α. (D) COX-derived- adrenic acid dihomo-PGE2. (E) COX-derived ARA oxylipins, PGE2, 15k-PGE2, 11β-PGE2, 20OH-PGE2, dhk-PGD2/E2. (F) COX-derived ARA oxylipins, PGD2, 15d-PGD2, PGF2α, 8-iso-PGF2α, 11β-PGF2α. (G) COX-derived ARA oxylipins PGJ2, 15d-PGJ2, 1a,1b-DIHOME-15-deoxy-PGJ2 and PGK2. (H) LOX-derived ARA oxylipin 5-oxoETE. Statistical significance was determined by ANOVA followed by Tukey's post hoc test. *p < 0.05, **p < 0.01, ***p < 0.001, **** p < 0.0001.

**Figure S15** LA, ALA, EPA and DHA oxylipin profiles, sEH expression and key fatty acids in colon. (A) LA and LOX-derived LA oxylipin 13-HODE. (B) CYP derived LA oxylipin 9,10-EpOME and 12,13-EpOME. (C) sEH-derived LA oxylipin 12,13-DIHOME. (D) sEH expression in colon mucosa. (E) 10-nitrooleate. (F) ALA and LOX-derived ALA oxylipins 9-HOTrE and 13-HOTrE. (G) EPA. (H) DHA and LOX-derived DHA oxylipin 13-HDHA. (I) Palmitoleic acid. Statistical significance was determined by ANOVA followed by Tukey's post hoc test. *p < 0.05, **p < 0.01, ***p < 0.001, **** p < 0.0001.

**Figure S16** Carnitine profile in the distal jejunum. (A) Butylcarnitine (C4:0). (B) Isovalerylcarnitine (3M-C4:0). (C) Hexanoylcarnitine (C6:0). (D) Decanoylcarnitine (C10:0). (E) Decenoylcarnitine (C10:1). (F) Dodecanoylcarnitine (C12:0). (G) Dodecenoylcarnitine (C12:1). (H) Myristoylcarnitine (C14). (I) Myristoleylcarnitine (C14:1). (J) Tetradecadienylcarnitine (C14:2). (K) Palmitylcarnitine (C16:0). (L) Palmitoleylcarnitine (C16:1). (M) Hexadecadienylcarnitine (C16:2). (N) Stearoylcarnitine (C18). (O) Oleylcarnitine (C18:1). (P) Linoleylcarnitine (C18:2). (Q) α-Linolenylcarnitine (C18:3). (R) Arachidonylcarnitine (C20:4). (S) Docosahexaenoylcarnitine (C22:6). Statistical significance was determined by ANOVA followed by Tukey's post hoc test. *p < 0.05, **p < 0.01, ***p < 0.001, **** p < 0.0001.

**Figure S17** Effects of oxidized olive oil on mitochondrial β-oxidation in the ileum. (A) Relative gene expression of PPARα, CPT1a, SCAD, MCAD and LCAD. (B) sPLS-DA plot showing the distribution of carnitines in the ileum. (B) Statistical differences in carnitine concentrations in distal jejunum among different diets. (C) Profile of carnitines in the ileum. (D) Statistical differences and fold change of carnitine concentrations in the ileum, HFD-ox-OO vs HFD. (E) Myristolycarnitine (C14), Stearoylcarnitine (C18) and Oleylcarnitine (C18:1). Data are presented as mean or mean ± SEM. Statistical significance was determined by ANOVA followed by Tukey's post hoc test, *p < 0.05, **p < 0.01, ***p < 0.001, **** p < 0.0001.

**Figure S18** Carnitine profile in the colon. (A) Propionylcarnitine (C3). (B) Butylcarnitine (C4). (C) Hexanoylcarnitine (C6). (D) Octanoylcarnitine (C8). (E) Octenylcarnitine (C8:1). (F) Decanoylcarnitine (C10). (G) Decenoylcarnitine (C10:1). (H) Decadienylcarnitine (C10:2). (I) Dodecenoylcarnitine (C12:1). (J) Myristoylcarnitine (C14). (K) Myristoleylcarnitine (C14:1). (L) Tetradecadienylcarnitine (C14:2). (M) Palmitylcarnitine (C16). (N) Palmitoleylcarnitine (C16:1). (O) Hexadecadienylcarnitine (C16:2). (P) Stearoylcarnitine (C18). (Q) Oleylcarnitine (C18:1). (R) Linoleylcarnitine (C18:2). (S) α-Linoleylcarnitine (C18:3). (T) Arachidonylcarnitine (C20:4). (U) Docosahexaenoylcarnitine (C22:6). Statistical significance was determined by ANOVA followed by Tukey's post hoc test. *p < 0.05, **p < 0.01, ***p < 0.001, **** p < 0.0001.

**Figure S19** Effects of oxidized olive oil on gut microbiota composition. (A) Chao 1 index and Shannon index. (B) The composition of colonic microbiota at phylum level. (C) The composition of colonic microbiota at class level. (D) The composition of colonic microbiota at order level. (E) The composition of colonic microbiota at family level.

**Figure S20** The effects of ox-OO olive oil on inflammatory response and lipid metabolism in white adipose tissue and liver. (A) Relative gene expression of COX2 in white adipose tissue. (B) Representative H&E-stained white adipose section. (C) COX-derived ARA oxylipins PGD2/E2 and 12-HHTrE. (D) LOX-derived LA oxylipin 13-HODE. (E) CYP derived LA oxylipin 9,10-EpOME and 12,13-EpOME. (F) sEH-derived LA oxylipin 12,13-DIHOME. (G) sEH-derived ALA oxylipins 12,13-DIHODE and 15,16-DIHODE. Statistical significance was determined by ANOVA followed by Tukey's post hoc test. *p < 0.05, **p < 0.01, ***p < 0.001, **** p < 0.0001.
