# Supplementary figures and images for: Dietary oxidized lipids in redox biology: Oxidized olive oil disrupts lipid metabolism and induces intestinal and hepatic inflammation in C57BL/6J mice
Source: Redox Biol. 2025 Mar 1;81:103575. doi: 10.1016/j.redox.2025.103575 (PMC11927754; doi:10.1016/j.redox.2025.103575)

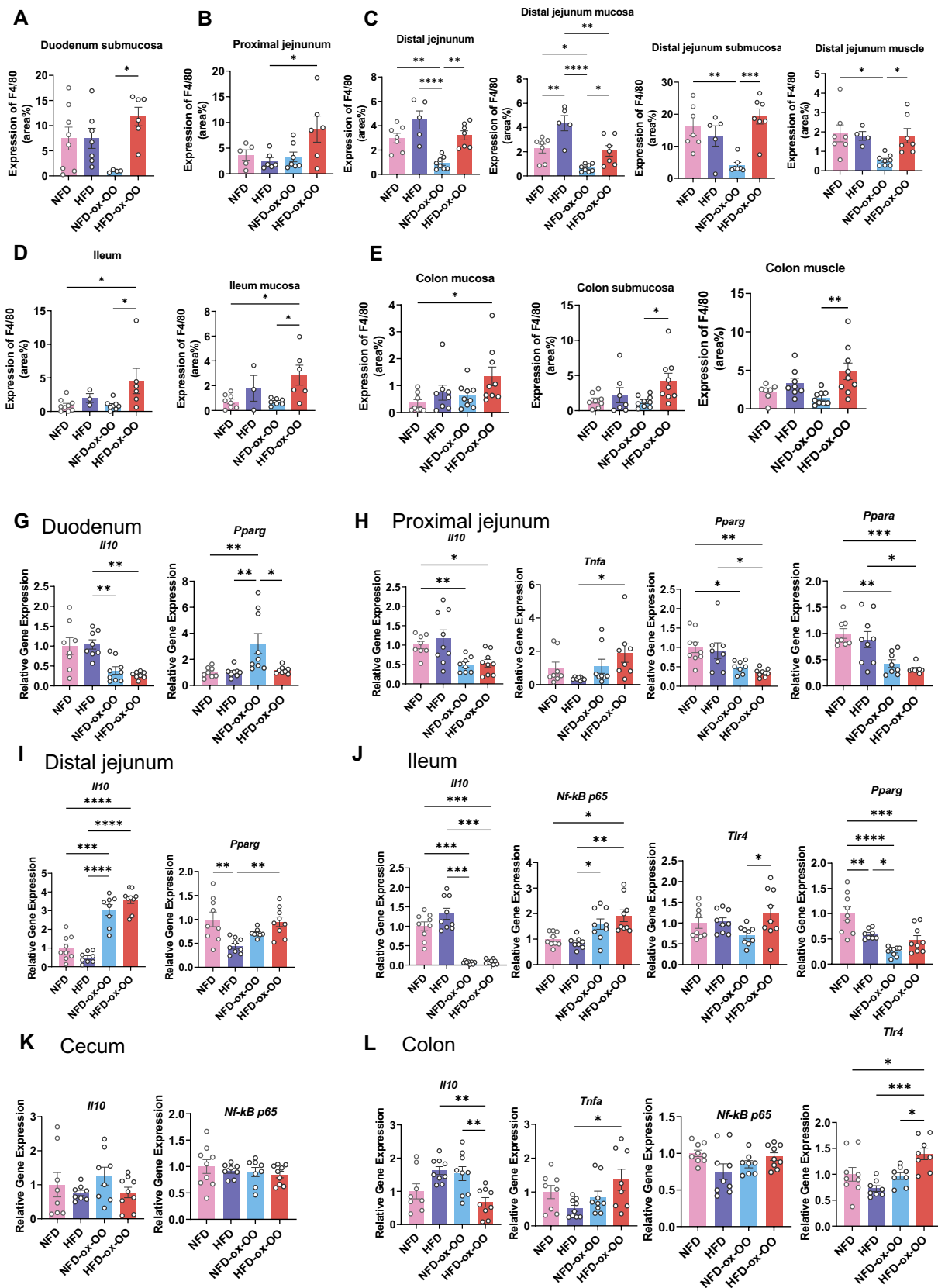

Supplement: Multimedia component 3 [file mmc3.pdf]

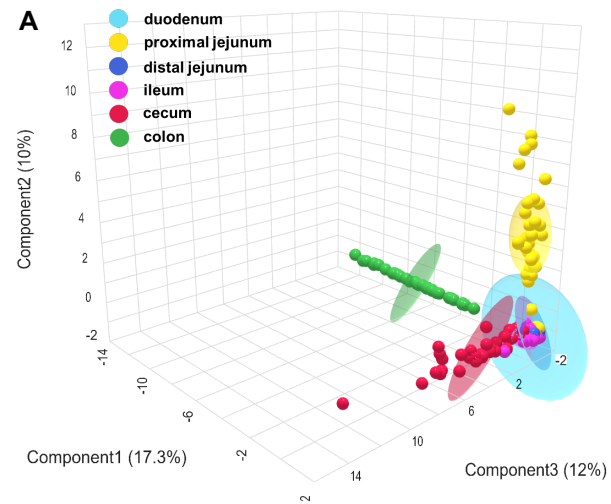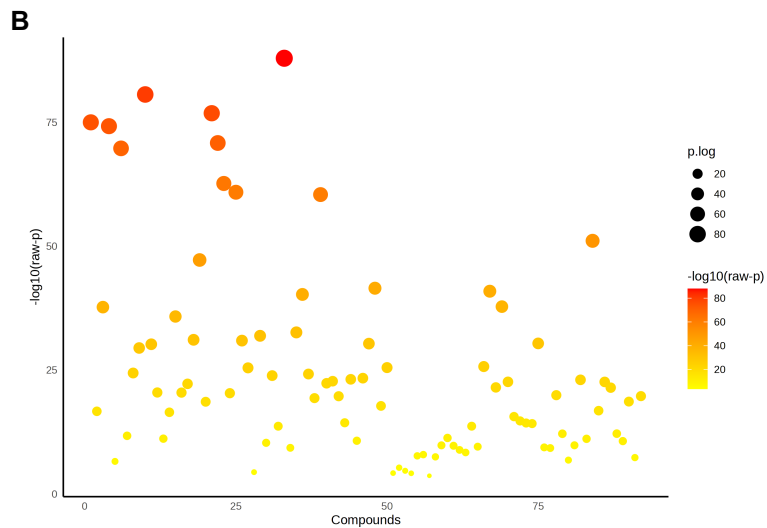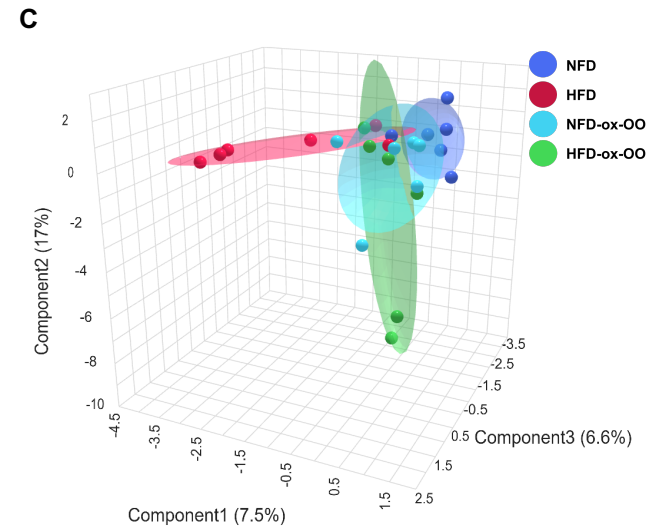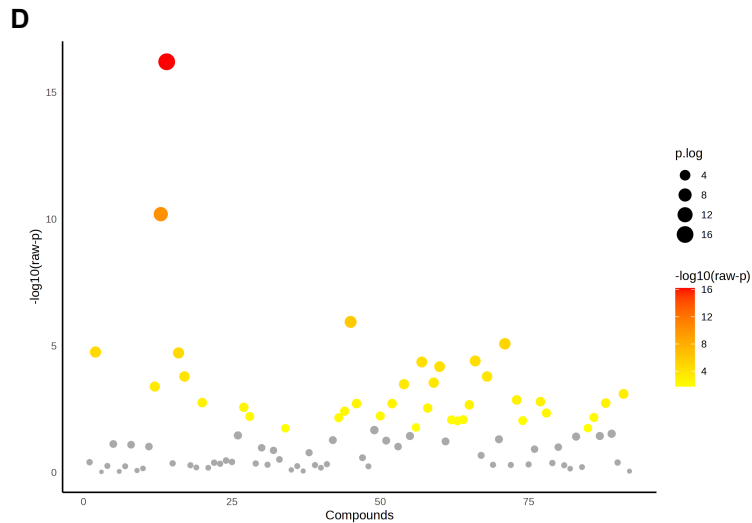

Supplement: Multimedia component 4 [file mmc4.pdf]

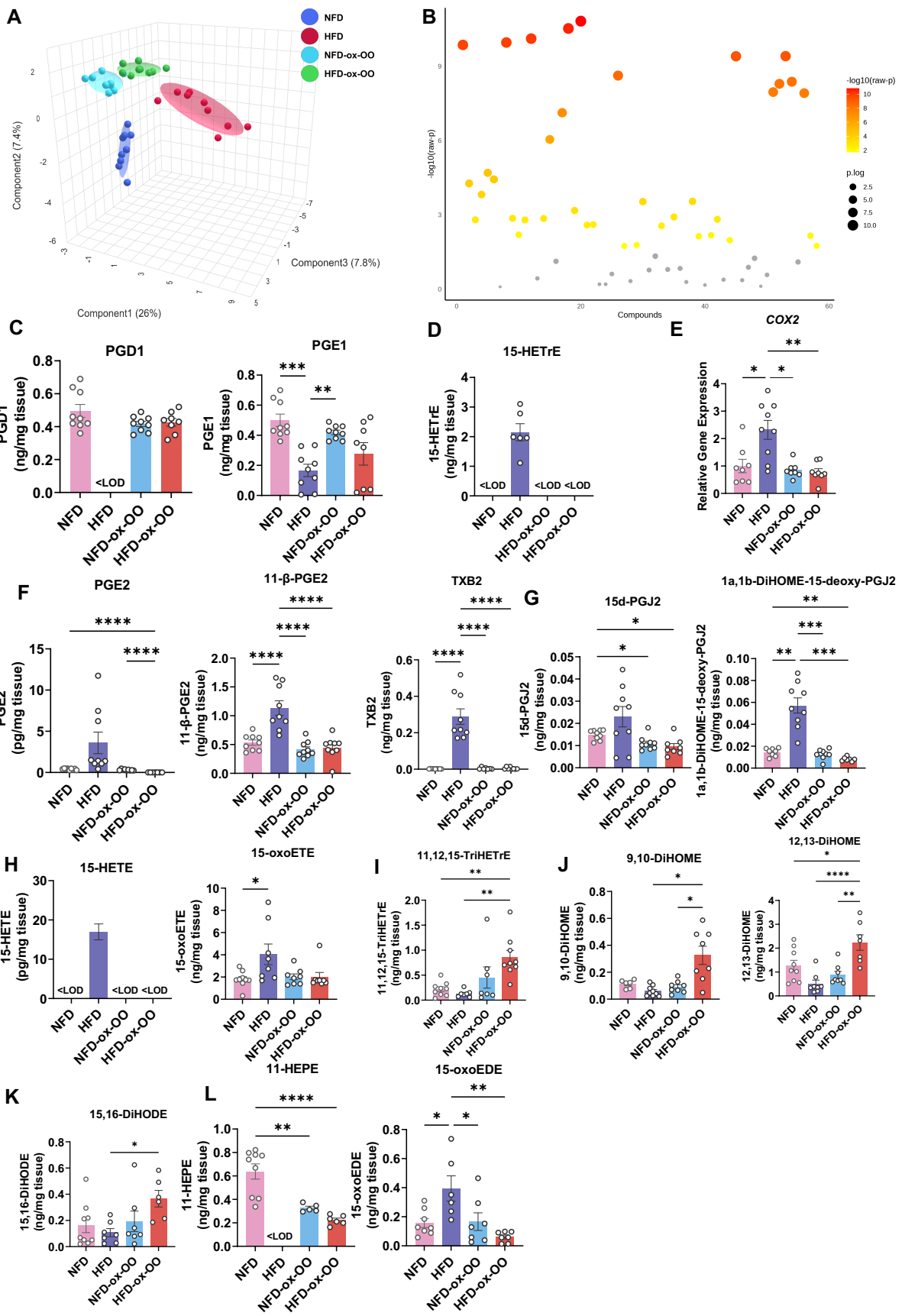

Supplement: Multimedia component 5 [file mmc5.pdf]

**A**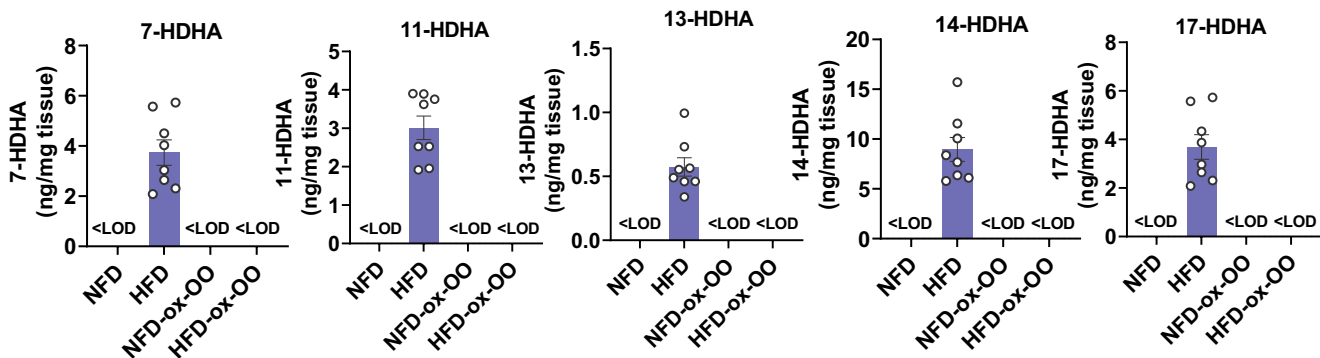**B**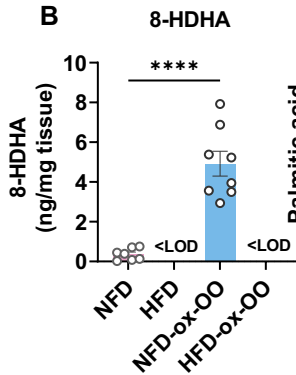**C**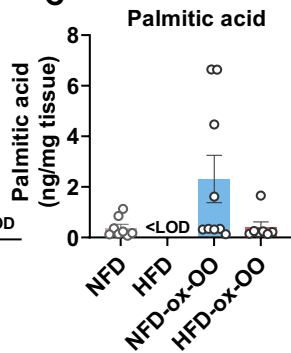**D**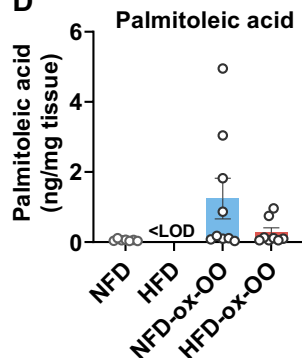

Supplement: Multimedia component 6 [file mmc6.pdf]

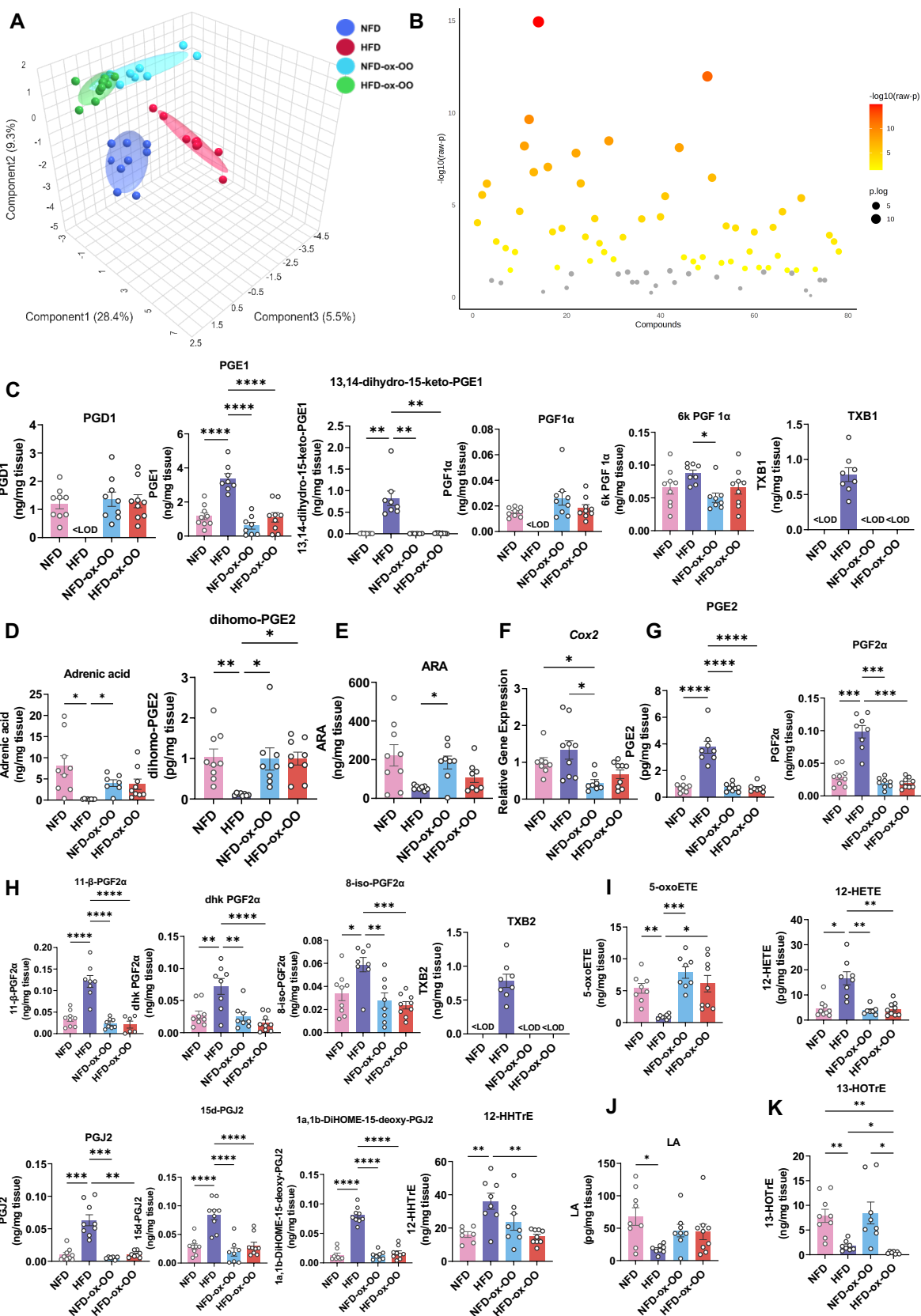

Supplement: Multimedia component 7 [file mmc7.pdf]

**A**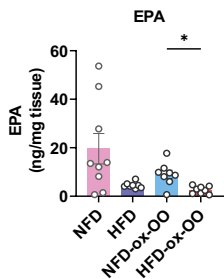**B**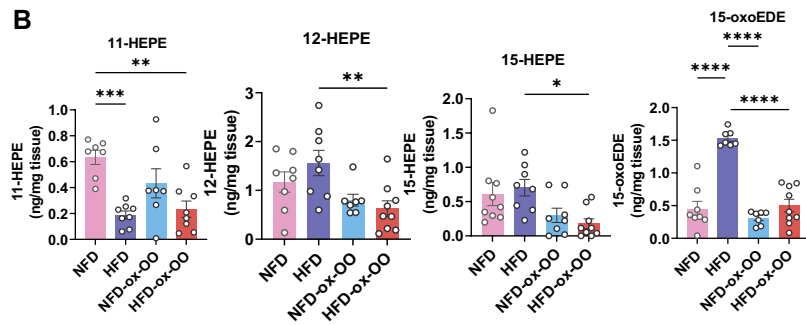**C**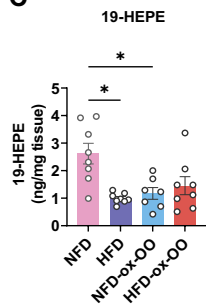**D**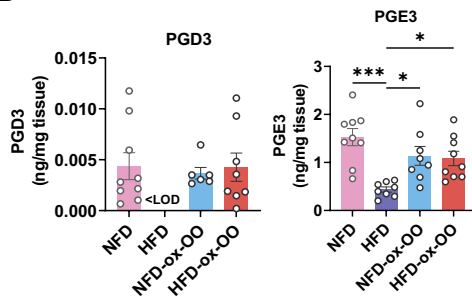**E**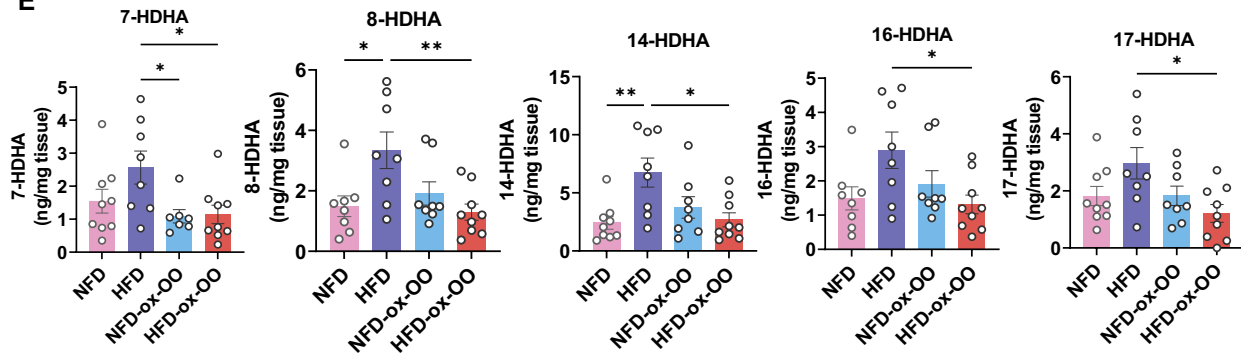**F**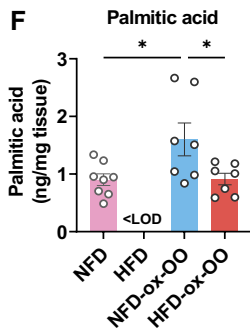**G**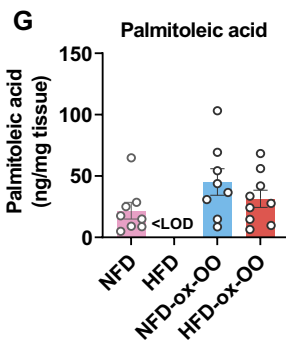**H**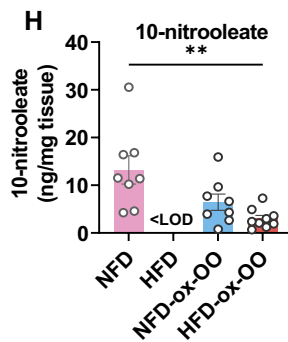

Supplement: Multimedia component 8 [file mmc8.pdf]

**A**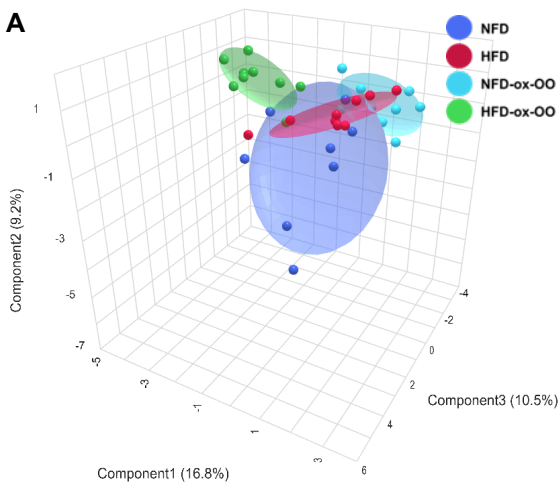**B**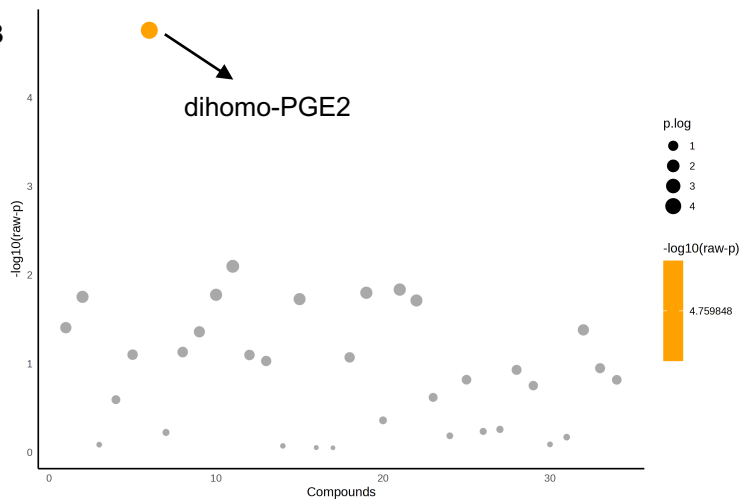**C**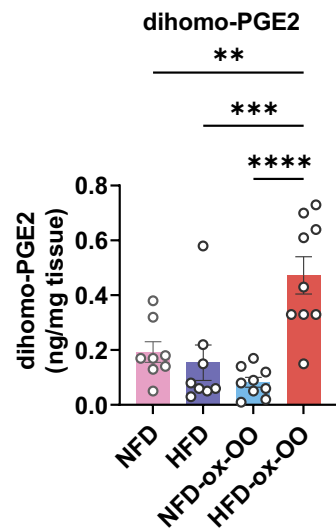**D**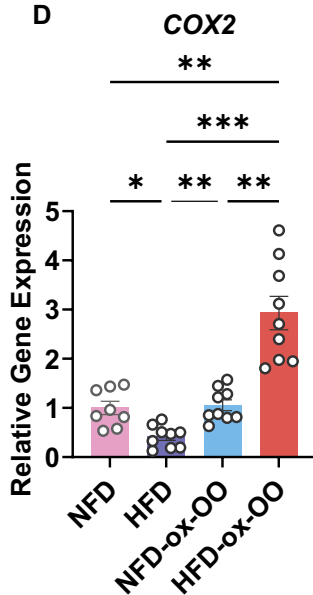

Supplement: Multimedia component 9 [file mmc9.pdf]

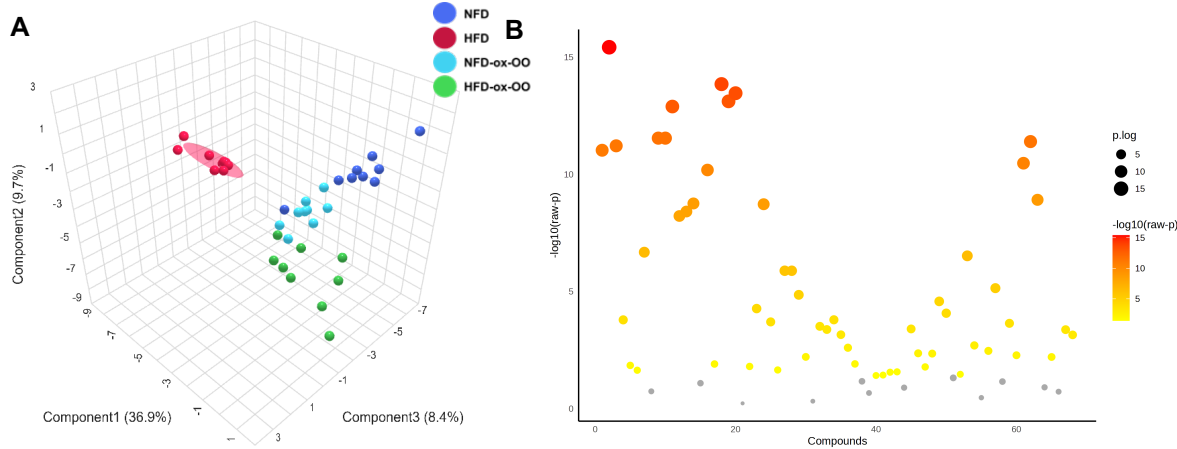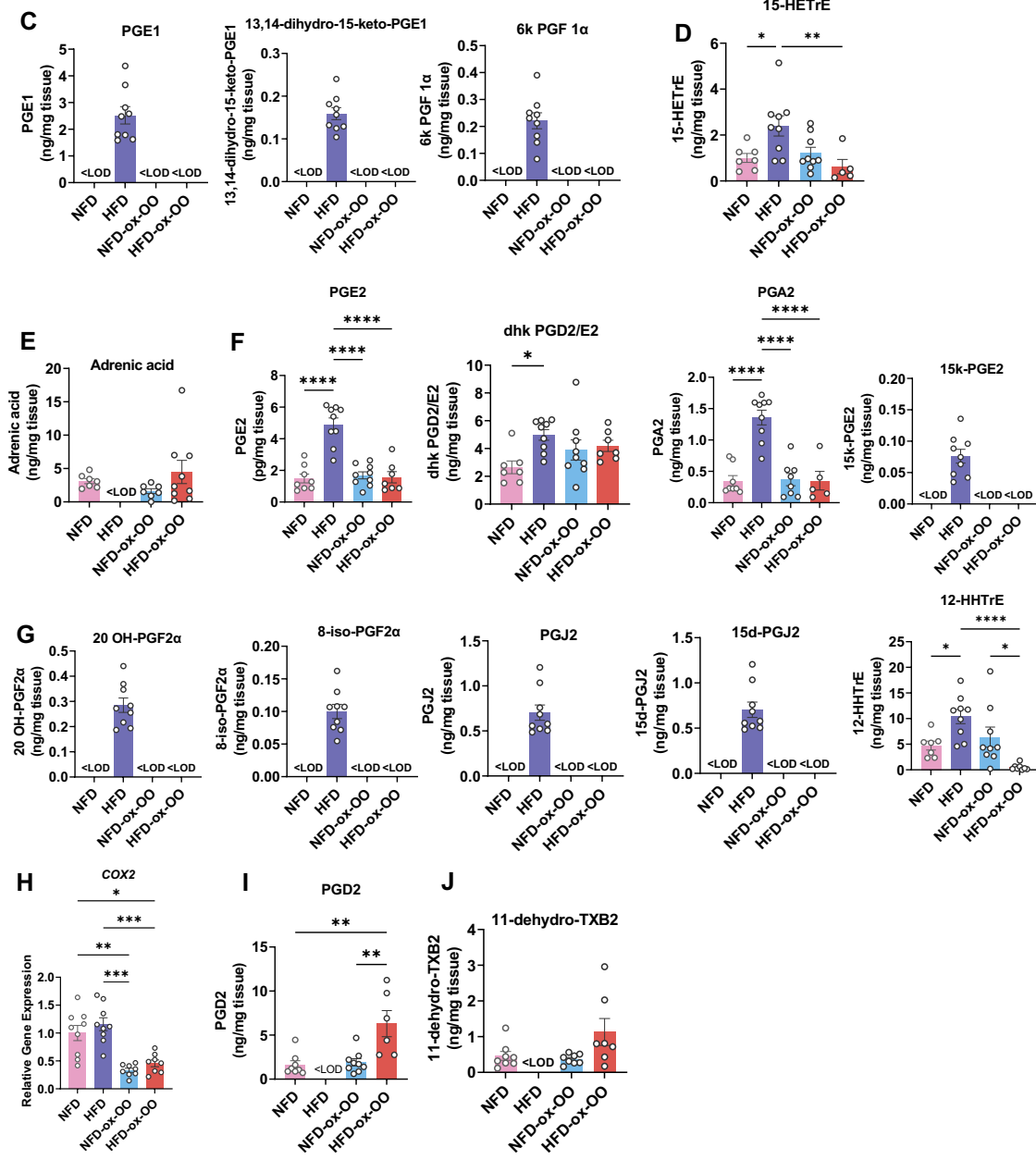

Supplement: Multimedia component 10 [file mmc10.pdf]

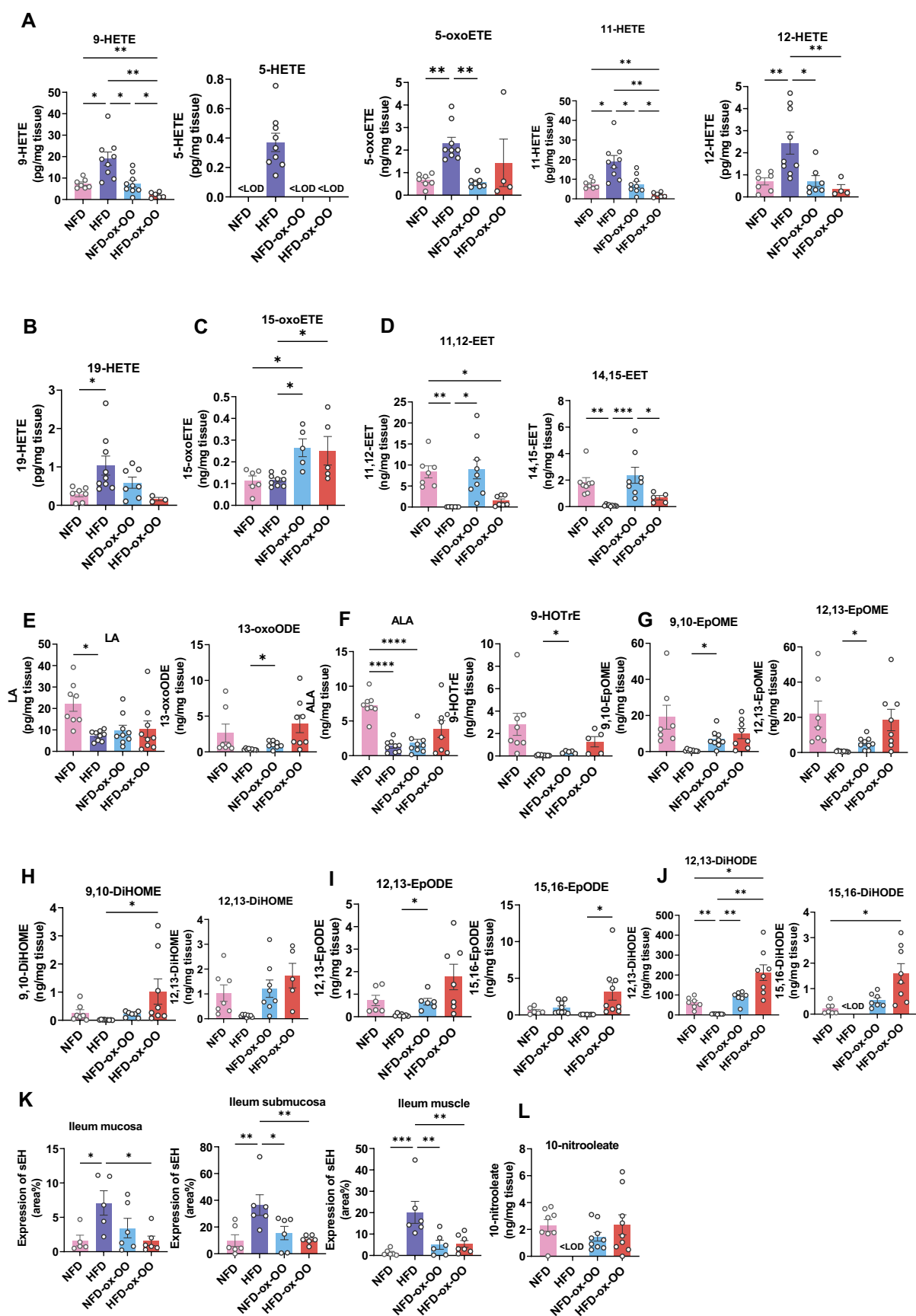

Supplement: Multimedia component 11 [file mmc11.pdf]

**A**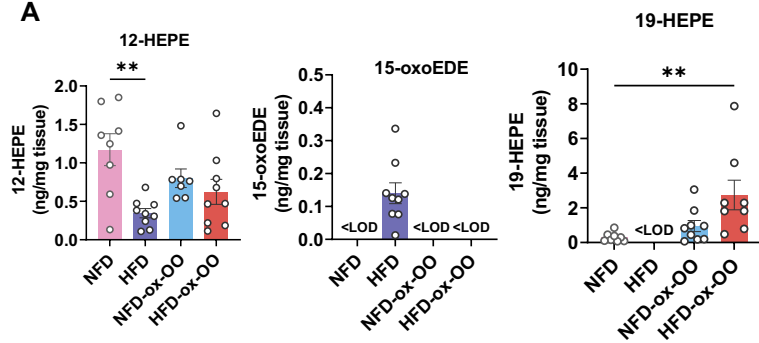**C**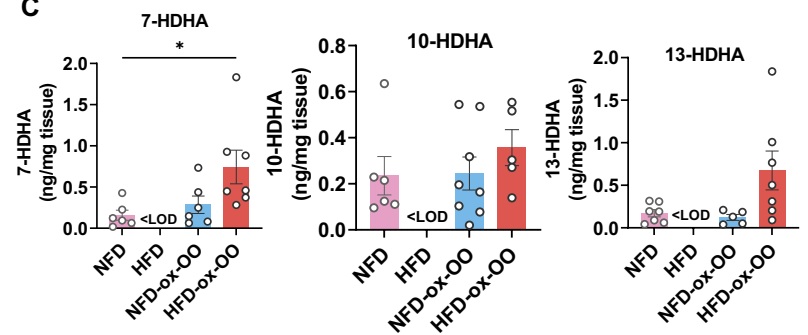**D**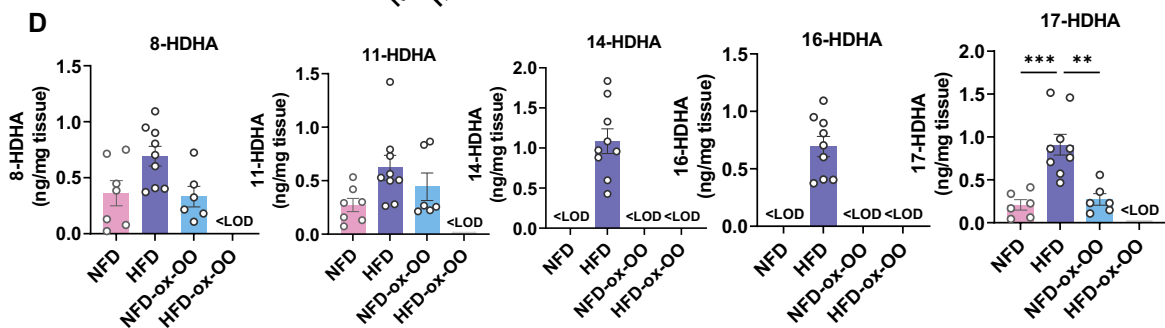**E**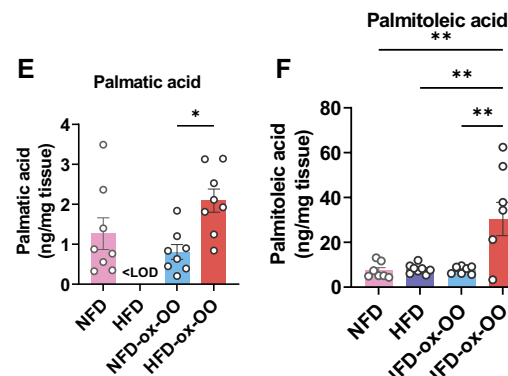**F**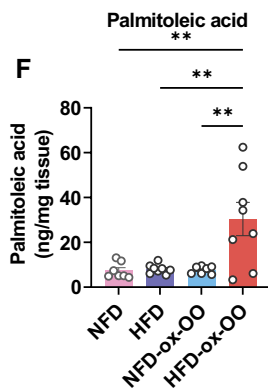

Supplement: Multimedia component 12 [file mmc12.pdf]

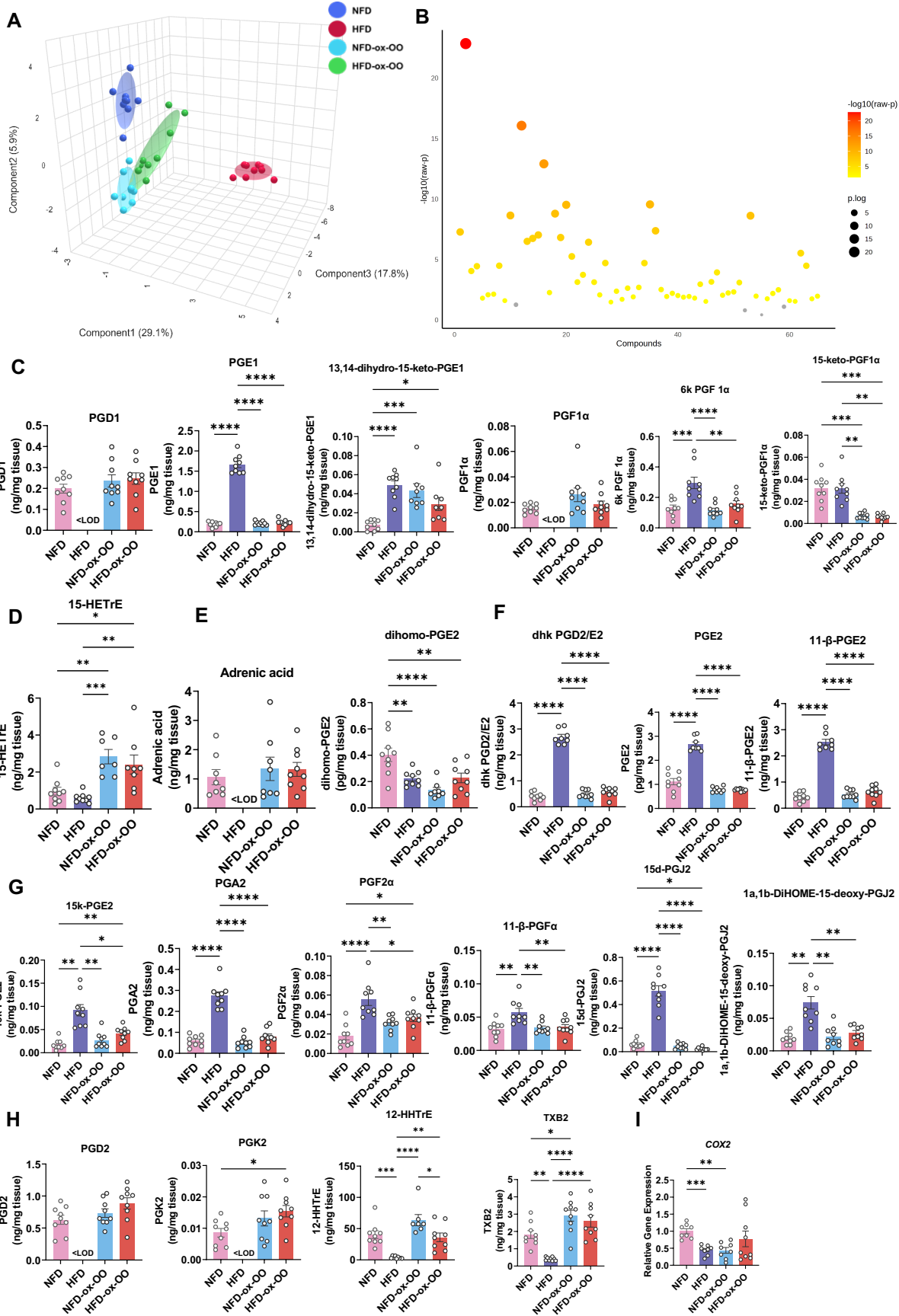

Supplement: Multimedia component 13 [file mmc13.pdf]

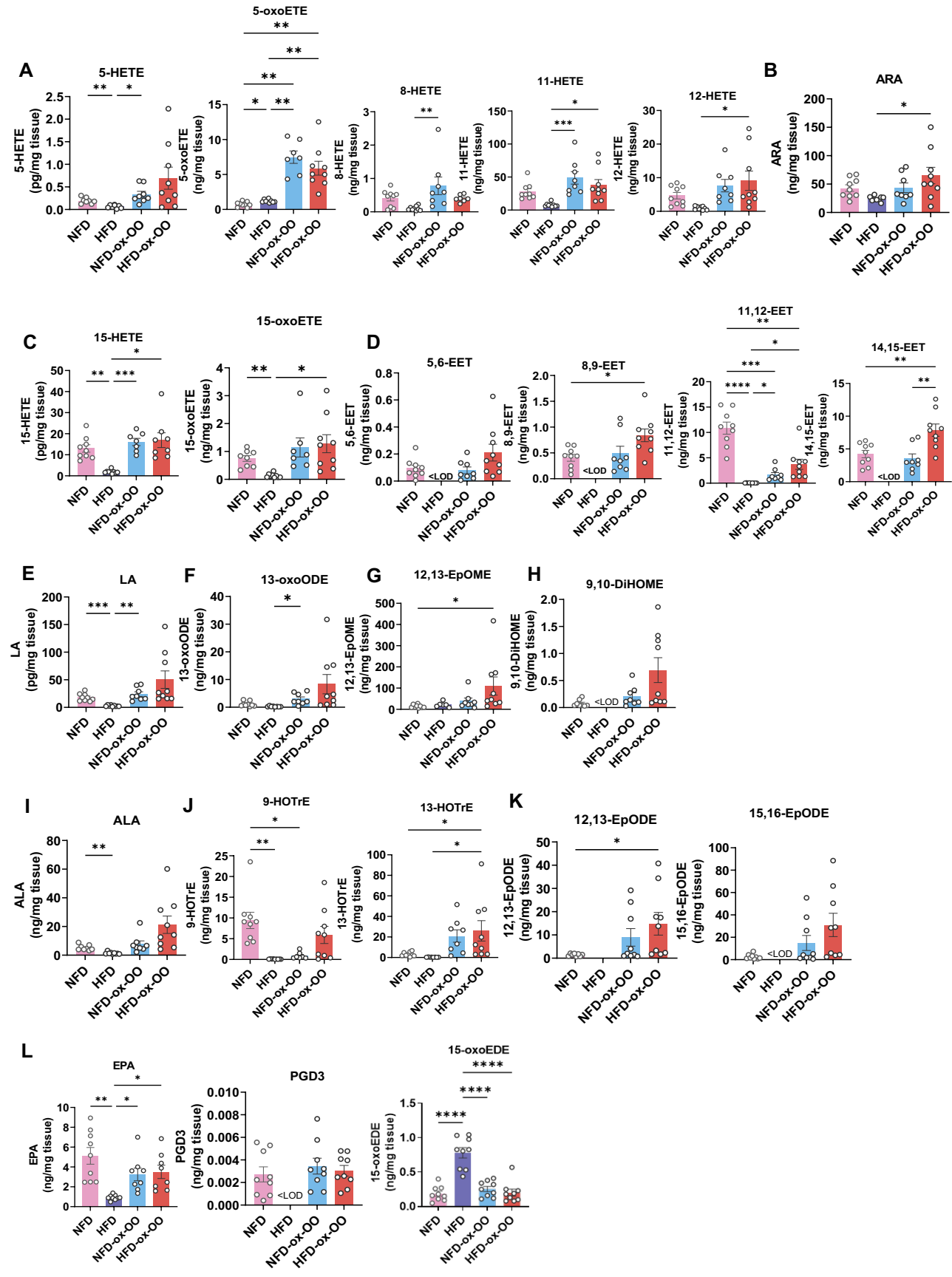

Supplement: Multimedia component 14 [file mmc14.pdf]

**A**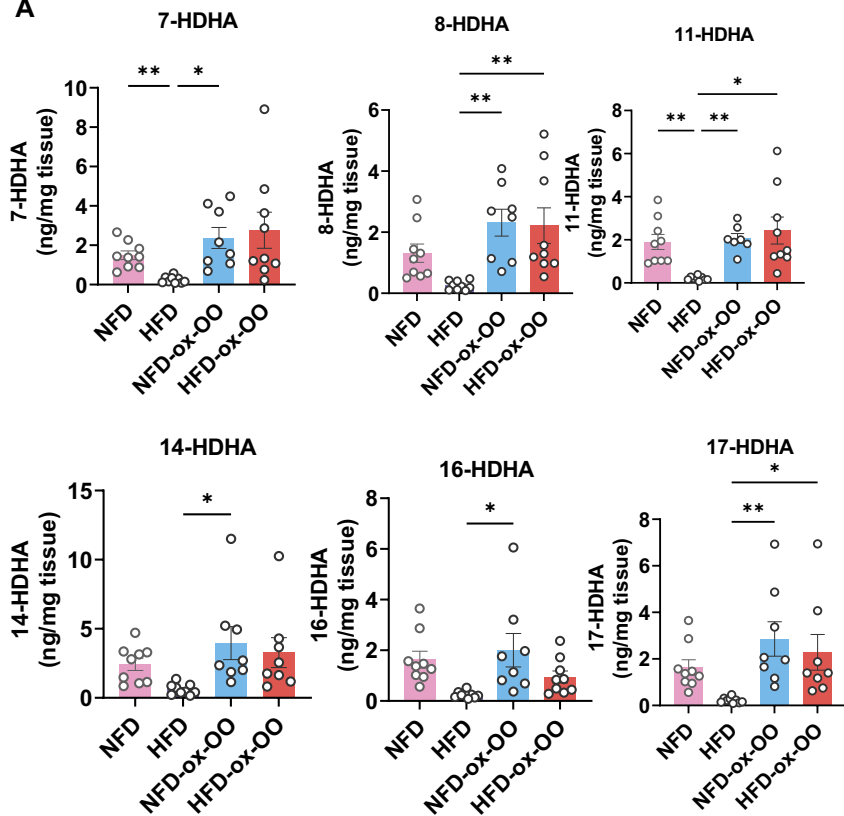**B**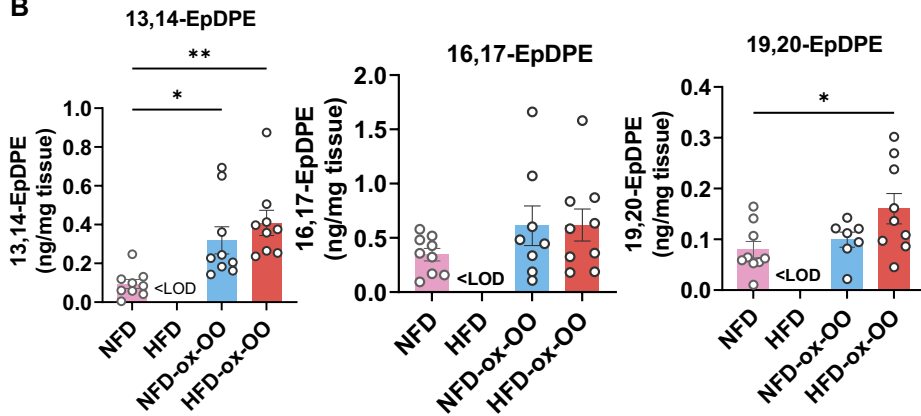**C**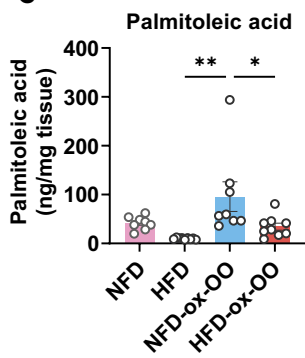

Supplement: Multimedia component 15 [file mmc15.pdf]

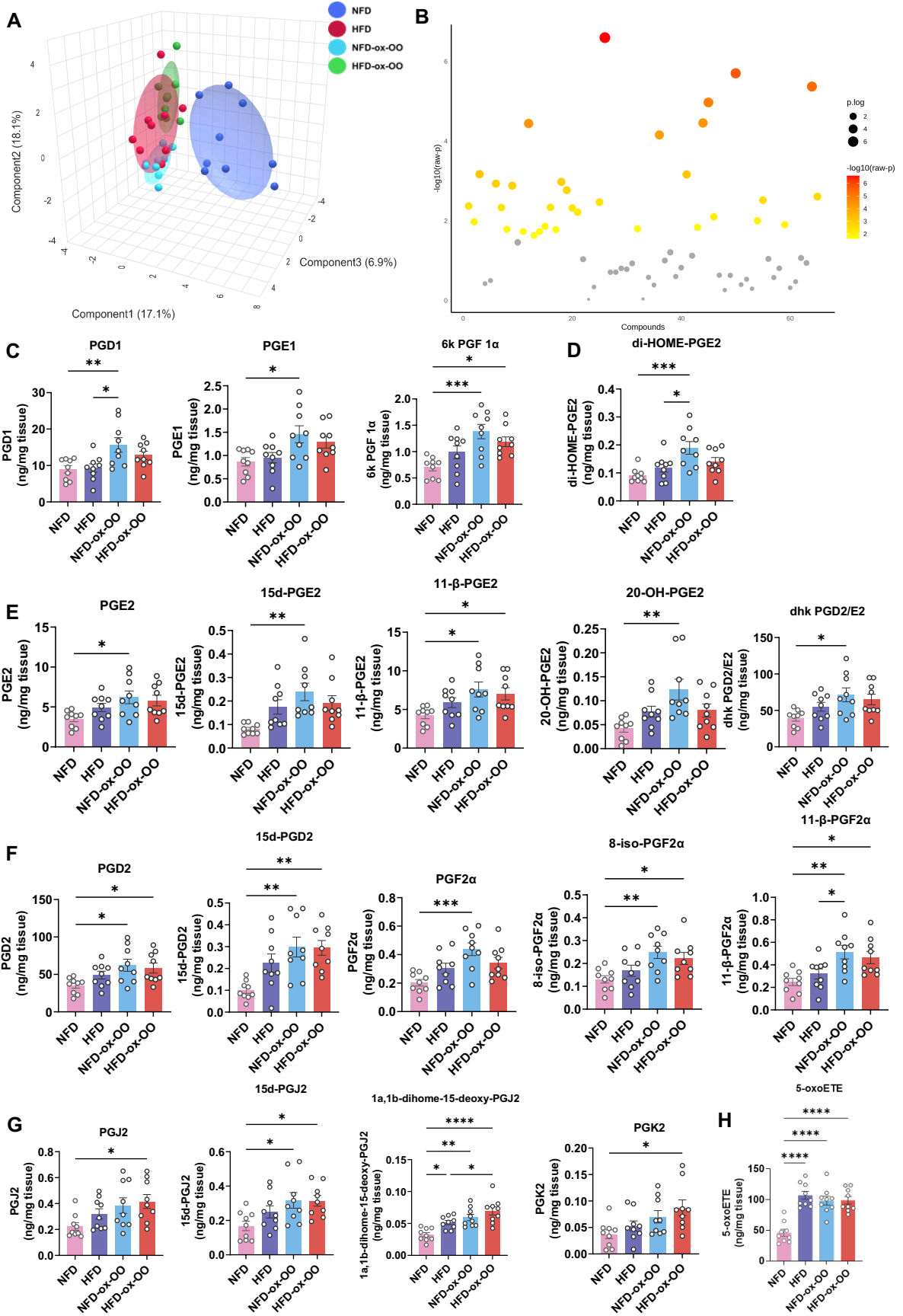

Supplement: Multimedia component 16 [file mmc16.pdf]

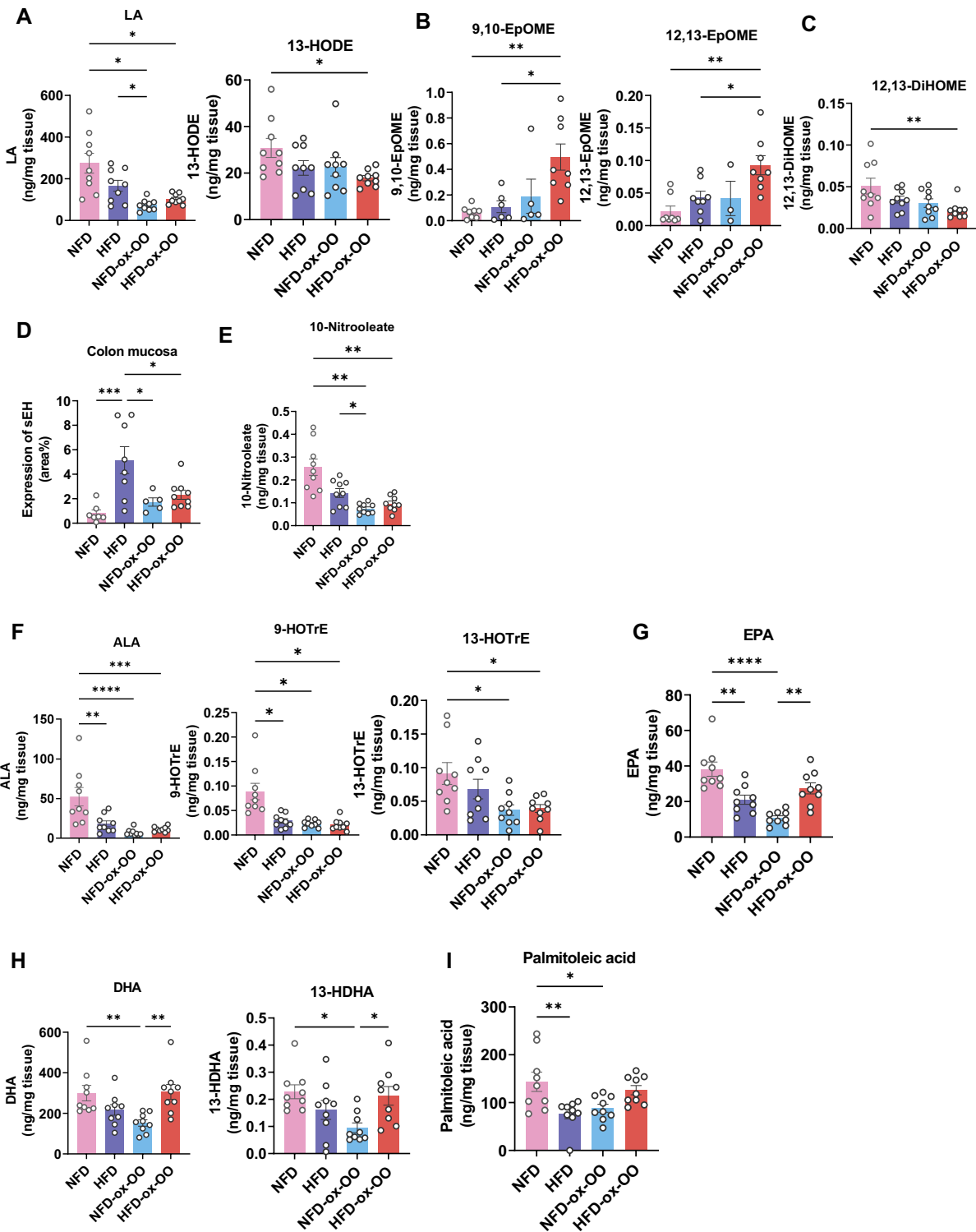

Supplement: Multimedia component 17 [file mmc17.pdf]

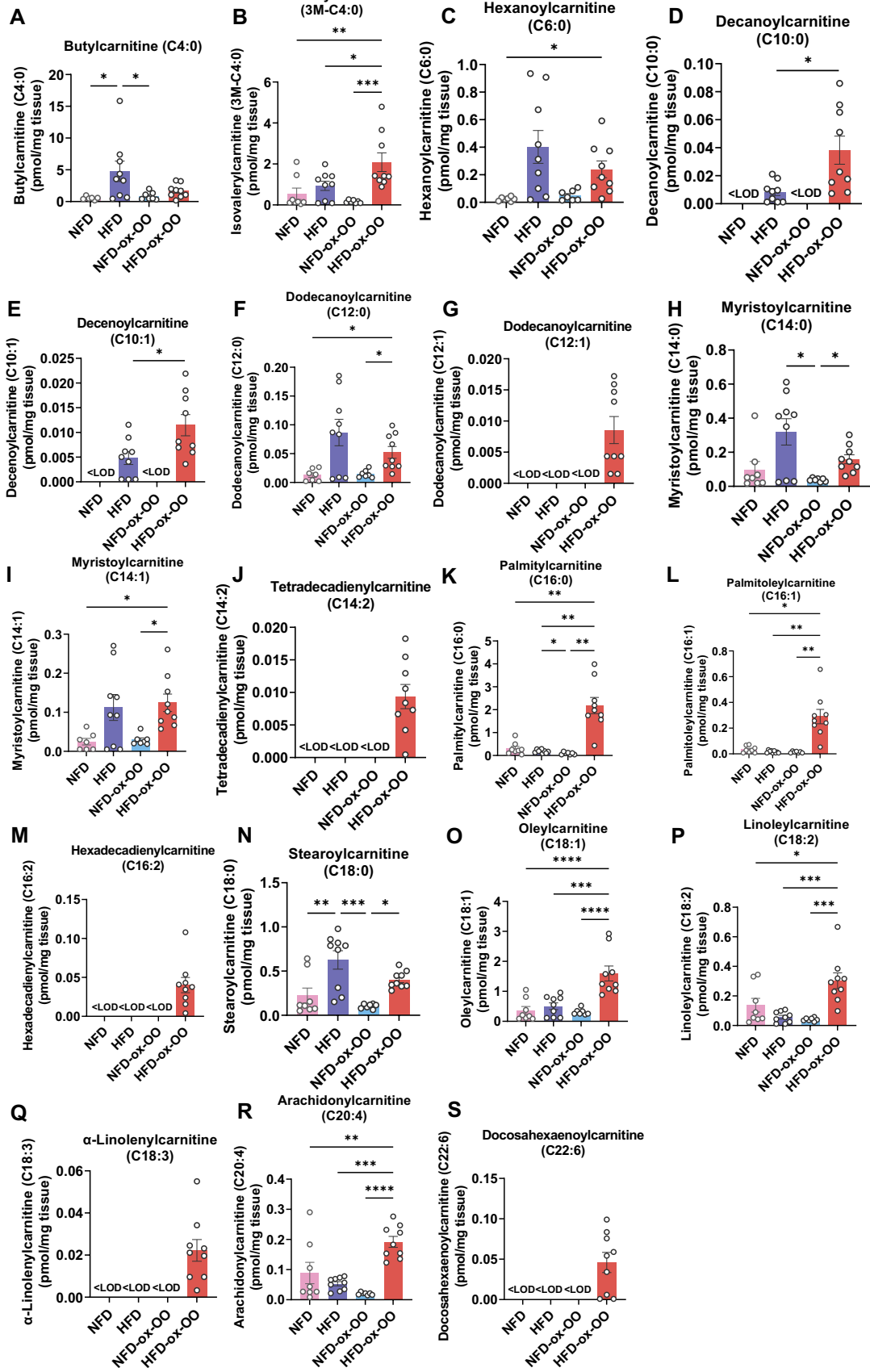

Supplement: Multimedia component 18 [file mmc18.pdf]

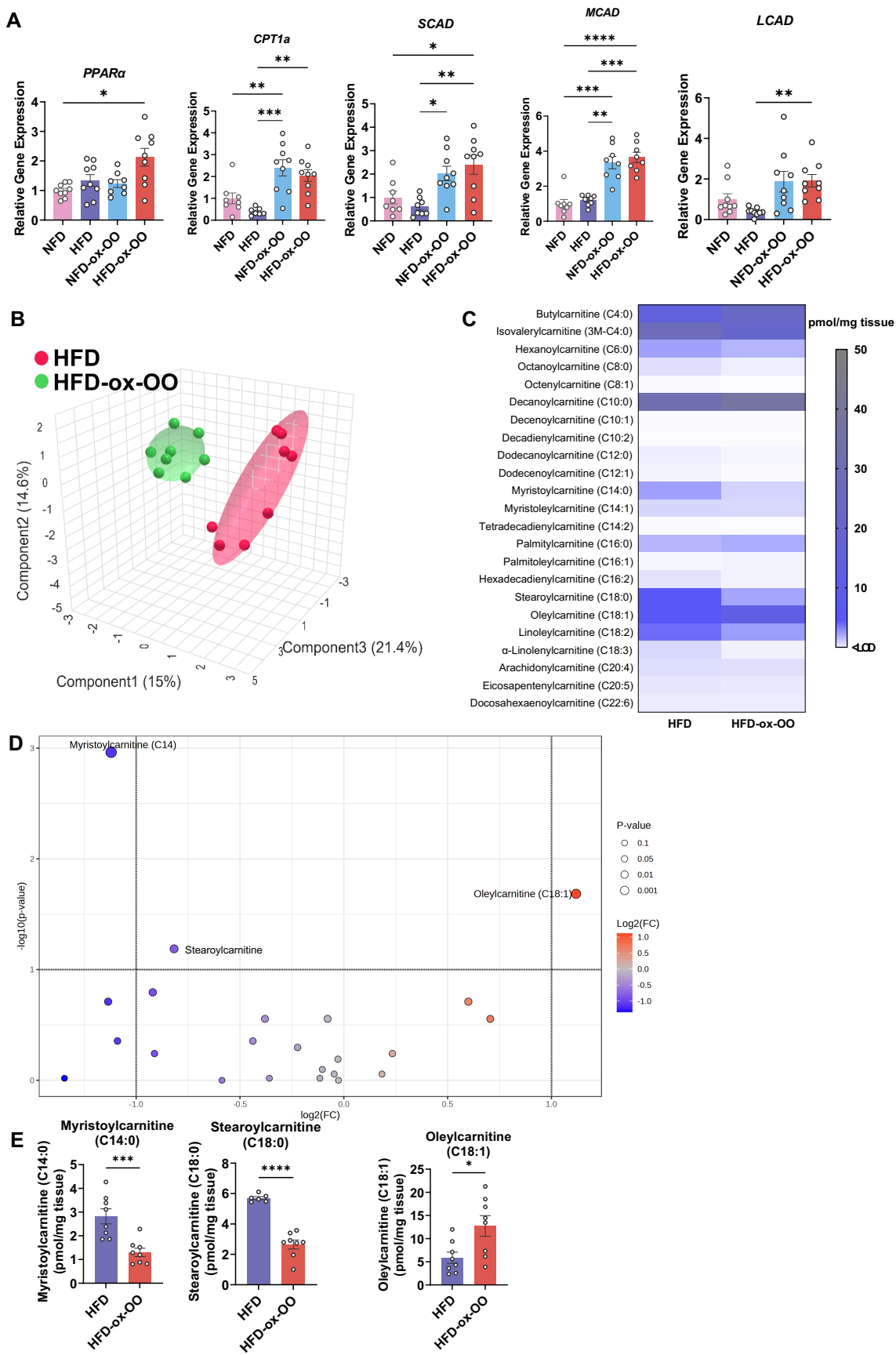

Supplement: Multimedia component 19 [file mmc19.pdf]

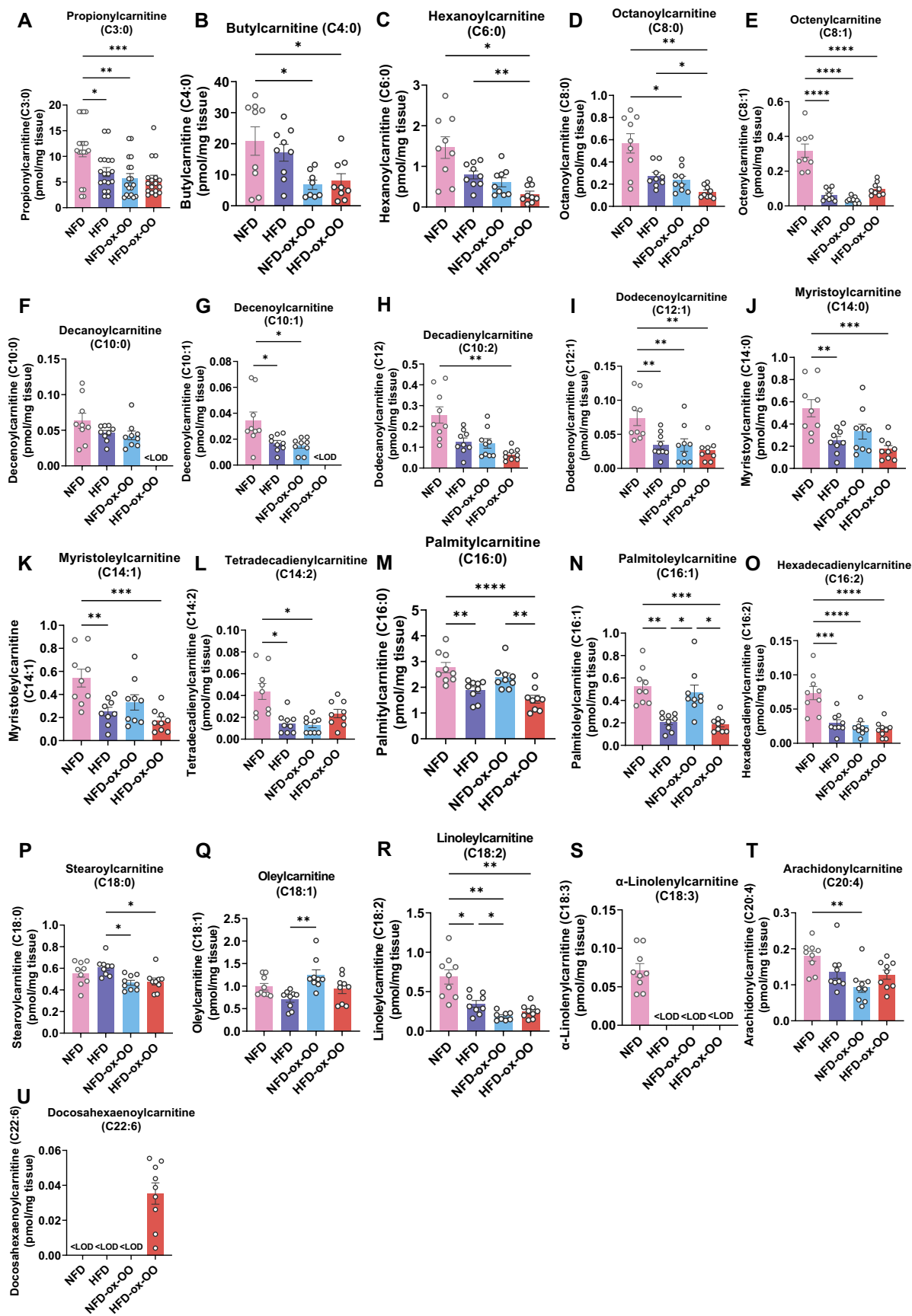

Supplement: Multimedia component 20 [file mmc20.pdf]

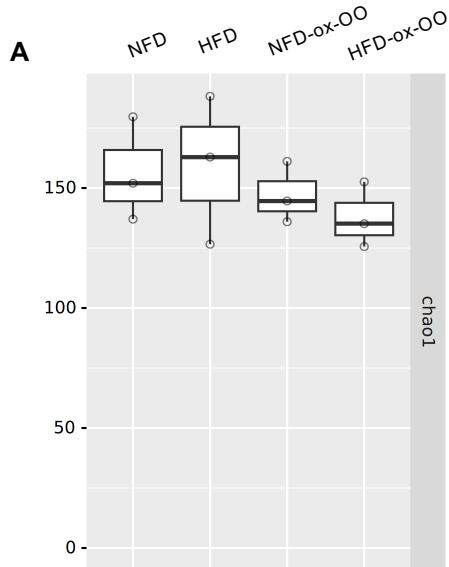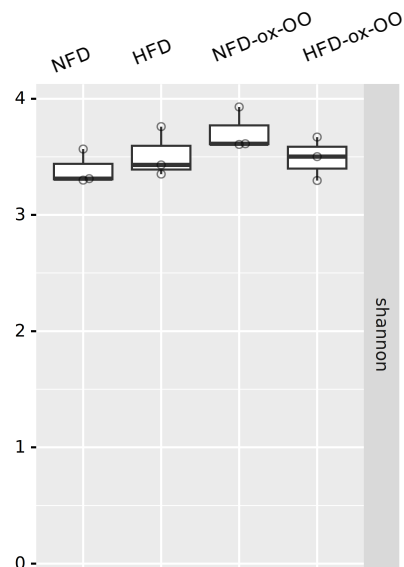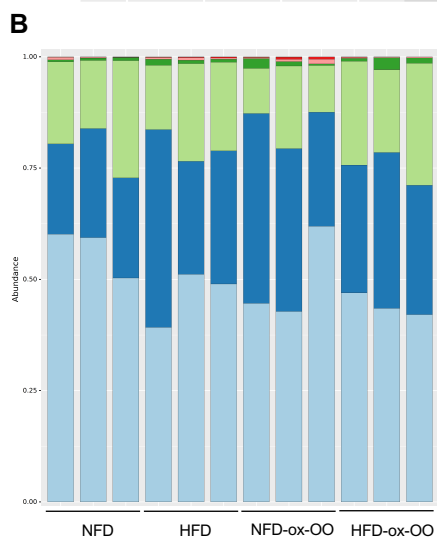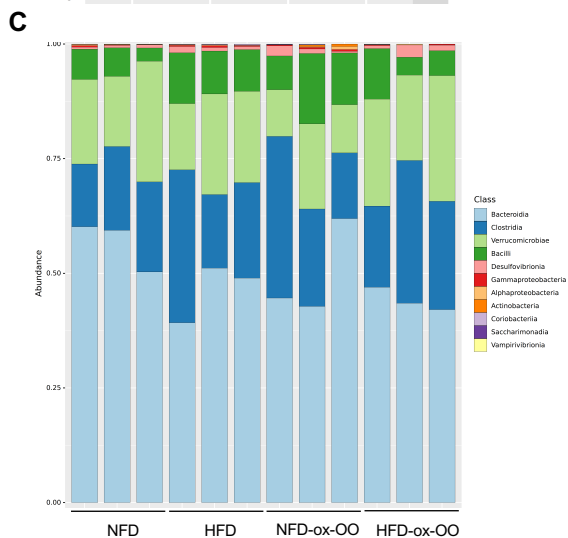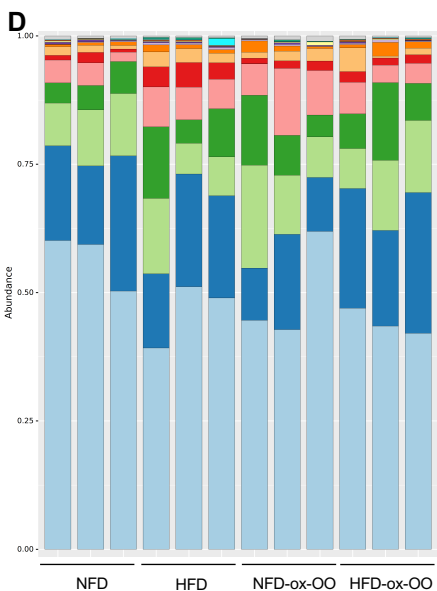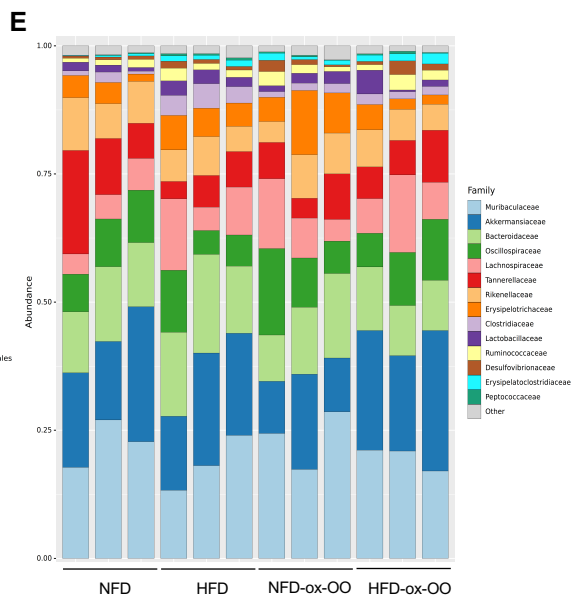

Supplement: Multimedia component 21 [file mmc21.pdf]

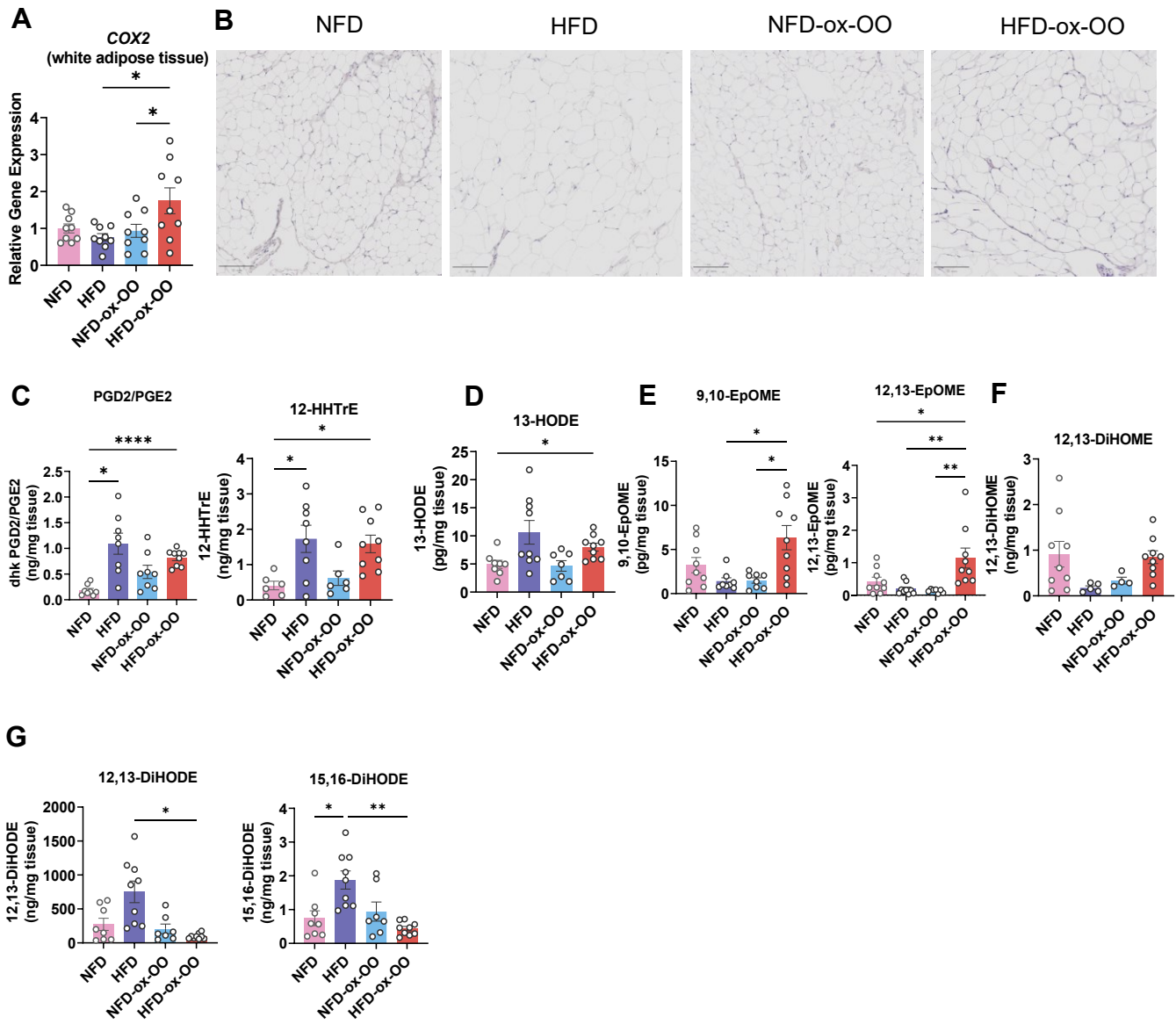

Supplement: Multimedia component 22 [file mmc22.pdf]
